# Emergency care interventions for paediatric severe acute respiratory infections in low- and middle-income countries: A systematic review and narrative synthesis

**DOI:** 10.7189/jogh.13.04065

**Published:** 2023-06-09

**Authors:** Pryanka Relan, Stephanie Chow Garbern, Gerard O’Reilly, Corey B Bills, Megan Schultz, Sean Kivlehan, Indi Trehan, Torben K Becker

**Affiliations:** 1 Department of Emergency Medicine, Emory Healthcare Network, Atlanta, Georgia, USA; 2 Department of Emergency Medicine, Warren Alpert Medical School of Brown University, Providence, Rhode Island, USA; 3 Emergency and Trauma Centre, The Alfred, Melbourne, Australia; School of Public Health and Preventive Medicine, Monash University, Melbourne, Australia; 4 Department of Emergency Medicine, University of Colorado School of Medicine, Aurora, Colorado, USA; 5 Department of Pediatrics, Medical College of Wisconsin, Milwaukee WI, USA; 6 Departments of Pediatrics, Global Health, and Epidemiology, University of Washington, Seattle, Washington, USA; 7 Department of Emergency Medicine, Brigham and Women's Hospital, Boston, MA, and Harvard Humanitarian Initiative, Cambridge, Massachusetts, USA; 8 Department of Emergency Medicine, University of Florida, Gainesville, Florida, USA

## Abstract

**Background:**

Severe acute respiratory infections (SARIs) are the leading cause of paediatric death globally, particularly in low- and middle-income countries (LMICs). Given the potential rapid clinical decompensation and high mortality rate from SARIs, interventions that facilitate the early care are critical to improving patient outcomes. Through this systematic review, we aimed to evaluate the impact of emergency care interventions on improving clinical outcomes of paediatric patients with SARIs in LMICs.

**Methods:**

We searched PubMed, Global Health, and Global Index Medicus for peer-reviewed clinical trials or studies with comparator groups published before November 2020. We included all studies which evaluated acute and emergency care interventions on clinical outcomes for children (29 days to 19 years) with SARIs conducted in LMICs. Due to observed heterogeneity of interventions and outcomes, we performed narrative synthesis. We assessed bias using the Risk of Bias 2 and Risk of Bias in Non-Randomized Studies of Interventions tools.

**Results:**

We screened 20 583, 99 of which met the inclusion criteria. Conditions studied included pneumonia or acute lower respiratory infection (61.6%) and bronchiolitis (29.3%). Studies evaluated medications (80.8%), respiratory support (14.1%), and supportive care (5%). We found the strongest evidence of benefit for decreasing risk of death for respiratory support interventions. Results were inconclusive on the utility of continuous positive airway pressure (CPAP). We found mixed results for interventions for bronchiolitis, but a possible benefit for hypertonic nebulised saline to decrease hospital length of stay. Early use of adjuvant treatments such as Vitamin A, D, and zinc for pneumonia and bronchiolitis did not appear to have convincing evidence of benefit on clinical outcomes.

**Conclusions:**

Despite the high global burden of SARI in paediatric populations, few emergency care (EC) interventions have high quality evidence for benefit on clinical outcomes in LMICs. Respiratory support interventions have the strongest evidence for benefit. Further research on the use of CPAP in diverse settings is needed, as is a stronger evidence base for EC interventions for children with SARI, including metrics on the timing of interventions.

**Registration:**

PROSPERO (CRD42020216117)

Acute respiratory infections (ARIs) are the leading cause of paediatric morbidity and mortality in low- and middle-income countries (LMICs), with pneumonia being the leading infectious cause of death in children under five years globally, causing over 800 000 deaths in 2019 [1,2]. ARIs encompass a broad range of conditions from a simple “cold” or upper respiratory infection to bronchiolitis, pneumonia, respiratory involvement of measles, and complications such as acute respiratory distress syndrome (ARDS) [3,4]. The World Health Organization (WHO) updates its initial 2011 definition of severe acute respiratory infection (SARI) in 2017 to standardise surveillance for influenza-like illness (ILI), defining it as “an acute respiratory infection with cough and fever which requires hospitalization” [5].

SARIs can be caused by a variety of pathogens, including viruses, bacteria, and parasites. Certain viral aetiologies of SARI have pandemic-causing potential, such as pandemic influenza A (H1N1/09) and severe acute respiratory syndrome coronavirus 2 (SARS-CoV-2). Children in LMICs are among the most vulnerable to poor outcomes from SARI due to inadequate immunisation coverage, underlying conditions such as malnutrition, and limited access to healthcare resources such as medical oxygen, ventilators, and relevant therapeutics. Emergency care (EC) interventions (i.e. those that provide or facilitate the early care for patients) are pivotal in improving outcomes for children with SARI.

Despite advances in characterising the aetiologies, incidence, and factors contributing to SARIs, identifying which EC interventions are most effective in improving clinical outcomes in LMICs is critical to ensuring that limited available resources can be optimally targeted towards feasible and effective interventions. Through this systematic review, we aimed to evaluate the impact of EC interventions on improving clinical outcomes of children with SARIs in LMICs.

## METHODS

We conducted this systematic review per the Preferred Reporting Items for Systematic Reviews and Meta-analyses (PRISMA) guidelines and in collaboration with the Global Emergency Medicine Literature Review (GEMLR) groups [6]. We registered it on PROSPERO (CRD42020216117). As we used only published, de-identified data, this study was exempt from institutional review board approval [7]. A full description of the systematic review methods has been previously published in the concurrently conducted adult-focused review and are summarised below [7].

### Search strategy and inclusion criteria

The full search strategy is available in Appendix S1 of the **Online Supplementary Document****.**

We searched PubMed, Global Health, and Global Index Medicus from November 2020 to January 2021. All randomised controlled trials (RCTs) and observational studies with a control group that evaluated short-term clinical outcomes and included each of three major search themes (SARI, emergency care interventions, and LMICs, as defined by the 2020 World Bank Classification) were eligible for inclusion. At least two independent reviewers (PR, SG, GO, CB, MS) screened each title and abstract followed by the full text of the remaining studies using Covidence, and a third reviewer (PR, SG, GO, CB, MS, TB, IT, SK) resolved any discrepancies. During the initial screening, we included studies with either paediatric and adult populations and later separated them into paediatric (29 days to 19 years, as per WHO definitions) and adult-focused reviews, given the variations in SARI epidemiology and management between adults and children. We excluded studies that focused exclusively on neonates (<28 days). We screened the studies based on the WHO SARI case definition criteria (i.e. regardless of whether the term SARI itself was used) due to a lack of consistent implementation of SARI surveillance across all LMIC contexts [7]. We defined EC interventions as relevant interventions initiated in the early period of care (within ~ 24 hours) without restrictions on setting. Only studies that evaluated patient-centric clinical outcomes such as mortality, need for intensive care unit (ICU) admission or mechanical ventilation, hospital length-of-stay (LOS), clinical severity, and symptom duration or severity were included.

### Assessment of risk of bias

Two authors (PR, SG) independently assessed the included studies for risk of bias using the Cochrane Risk of Bias 2 (RoB 2) tool for randomised trials and the Risk of Bias in Non-Randomized Studies (ROBINS-I) tool for observational studies; discrepancies were resolved by a third author. Risk of bias plots were created using the *robvis* package in R [8].

### Data extraction and analysis

Two authors (PR, SG) independently evaluated each article and extracted the data on a standardised form using Covidence, while a third author resolved any discrepancies. Due to the heterogeneity of interventions, settings, and outcomes measured, we could not conduct a meta-analysis, so we performed a qualitative analysis and narrative synthesis.

## RESULTS

**Figure 1:**
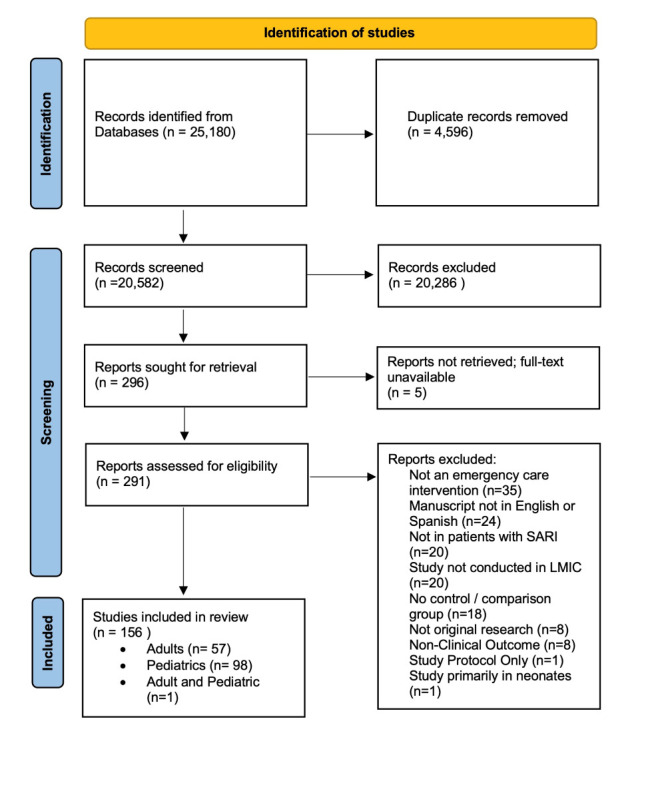
PRISMA flow diagram for systematic review.

**Figure 2:**
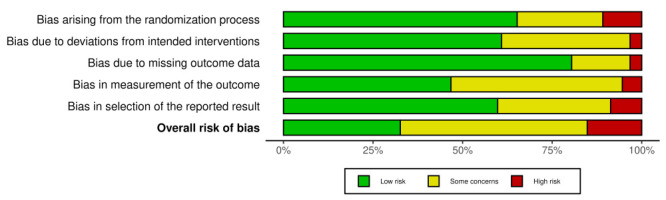
Summary of risk of bias assessments for included RCTs using the Cochrane RoB 2 tools.

The initial literature search retrieved 25 180 studies, with 20 223 remaining after deduplication. After title, abstract, and full-text screening, 99 studies met the inclusion criteria for further analysis (**Figure 1**). Pneumonia was the most common condition studied (52.5%), followed by bronchiolitis (29.3%) (**Table 1**). Medications were the most common intervention type (80.8%) followed by respiratory support (14.1%). Most studies were conducted in general hospital/paediatric wards (72.7%), while only 18.1% of studies enrolled patients in emergency wards (**Table 1**). Given the substantial number of studies, the results are presented by type of intervention, followed by the disease/condition studied (Table S1-S2 in the **Online Supplementary Document**). Results obtained from the risk of bias assessment for RCTs and for observational studies are summarised in **Figure 2** and Figures S1-S3 in the **Online Supplementary Document**.

**Table 1:** Characteristics of studies (n = 99)

	Number of studies (n = 99), n (%)
**Type of study**	
RCT	91 (91.9)
Observational, prospective	4 (4.0)
Observational, retrospective	3 (3.0)
Non-randomised trial	1 (1.0)
**World Bank income group**	
LMIC only	60 (60.6)
UMIC only	23 (23.2)
LIC only	12 (12.1)
Mixed: LIC, LMIC, UMIC	4 (4.0)
**WHO region**	
Southeast Asian	25 (25.3)
Eastern Mediterranean	24 (24.2)
African	21 (21.2)
American	14 (14.1)
Western Pacific	12 (12.1)
Mixed	2 (2.0)
European	1 (1.0)
**Disease/condition**	
Pneumonia	52 (52.5)
Bronchiolitis	29 (29.3)
ALRTI	9 (9.1)
Undifferentiated acute respiratory distress	5 (5.1)
Croup	2 (2.0)
Influenza	1 (1.0)
SARI	1 1.0)
**Intervention**	
Medications	80 (80.8)
Respiratory support	14 (14.1)
Supportive care	5 (5.0)
**Location where study took place**	
Emergency department	18 (18.1)
Hospital ward	72 (72.7)
Intensive care unit	3 (3.0)
Outpatient/other/not specified	6 (6.0)

RCT – randomised controlled trial, LIC – low-income country, LMIC – low- and middle-income country, UMIC – upper-middle income country, WHO – World Health Organization, ALRTI – acute lower respiratory tract infection, SARI – severe acute respiratory illness

### Respiratory support

Fourteen studies evaluated respiratory support interventions, including eight focused on oxygen delivery interventions and six on continuous positive airway pressure (CPAP).

#### Oxygen delivery, excluding CPAP

Eight studies evaluated various oxygen delivery systems, six on pneumonia or acute lower respiratory tract infection (ALRTI) and two on undifferentiated acute respiratory distress. Duke et al. [9] conducted a prospective cohort study in Papua New Guinea which found decreased mortality (but not statistically significant) among children monitored with pulse oximetry and administered oxygen if SpO_2_<85%. In their second study [10], implementation of an oxygen administration guideline and oxygen concentrators was significantly associated with 35% lower risk of death. Mulondo et al. [11] found that children with pneumonia treated with an oxygen-sparing nasal reservoir cannula had higher mean SpO_2_ without adverse events, although other clinical outcomes were not measured in small pilot RCT. Three RCTs among children with ALRTI were included; Muhe et al. [12,13] conducted two RCTs on oxygen administration via nasal catheter vs nasal prongs and found no differences in hypoxia or complications in children with ALRTI, although the authors recommended the use of nasal prongs as needed and nose bleeding/ulceration was greater for nasal catheter use. In a stepped-wedge cluster randomised trial, Graham et al. [14] found decreased odds of death among children with ALRTI treated in hospitals after implementation of multifaceted standardised oxygen system package (improved oxygen equipment, clinical education, technical training/support, infrastructure support) over pulse oximetry introduction or baseline (usual care prior to introducing pulse oximetry), although benefit was not seen in children overall.

Two studies evaluated interventions for undifferentiated respiratory distress; in a pre-post study, Hoffman et al. [110] (South Africa) found that children treated during a period where high-flow nasal cannula was available may have reduced need for transfers to higher level health facilities, although the study was retrospective and underpowered for mortality benefit. Kumar et al. [46] (India) evaluated several modes of oxygen delivery and found improved treatment of hypoxia among children administered oxygen via a headbox compared to face mask or nasal catheter.

#### CPAP, including bubble CPAP

Two RCTs evaluated bubble continuous positive airway pressure (bCPAP) for pneumonia. Chisti et al. found that, among children <5 years old with severe pneumonia and hypoxaemia in Bangladesh, those who received bCPAP had lower mortality rate (4% vs 15%; risk ratio = 0.25; 95% confidence interval (CI) = 0.07-0.89) and lower treatment failure compared to low-flow oxygen therapy [15]. However, McCollum et al. [16] found that bCPAP was associated with higher hospital mortality (17% vs 11%; RR = 1.52, 95% CI = 1.02-2.27) compared with low flow oxygen in children aged 1-29 months old with severe pneumonia and either HIV infection or exposure, severe malnutrition, or hypoxaemia, suggesting that bCPAP might carry previously unrecognised risks. Three studies evaluated bCPAP for undifferentiated respiratory distress; an RCT by Wilson et al. [17] in Ghana found decreased respiratory rate by 16 breaths/min in children treated with immediate initiation (<1 hour) of CPAP and no change in children who had CPAP delayed over an hour. Another RCT by Wilson et al. [18] found no difference in mortality at two weeks in unadjusted analysis, yet found a benefit with decreased mortality among children <1 year of age. Machen et al. [19] evaluated bCPAP in a non-randomised study among children with undifferentiated acute respiratory distress in Malawi and found the best outcomes among children with bronchiolitis (92.9% survival), followed by pneumonia (53.3% survival), and *pneumocystis jirovecii* (PJP) pneumonia (38.1% survival), but the study was limited by a lack of randomisation and comparison of clinical outcomes. Lal et al. [20] found improved respiratory rate and clinical respiratory distress scores (Silverman-Anderson and Modified Pediatric Society of New Zealand scores) at one hour in children with bronchiolitis treated with nasal CPAP compared to oxygen through mask or hood in India.

### Supportive care

Five studies evaluated supportive care interventions, including four on chest physiotherapy and one on steam therapy.

#### Physiotherapy

Four RCTs evaluated the effectiveness of chest physiotherapy (PT); two for pneumonia and two for bronchiolitis [21-24]. Lukrafka et al. [22] and Bohe et al. [23] found no differences in severity score or hospital LOS among children with pneumonia treated with chest PT. However, Abdelbasset et al. [21] (Egypt) found faster time to clinical resolution of pneumonia and improvement in respiratory rate in children in the chest PT group, while Gomes et al. [24] (Brazil) found improvement in Wang respiratory score in infants with bronchiolitis treated with “new” chest physical therapy (with prolonged slow expiration and clearance rhinopharyngeal retrograde) or conventional chest PT compared with suction of upper airways alone.

#### Other supportive care interventions

Singh et al. [25] found that steam therapy had no benefit in children with pneumonia compared to standard of care, although improvement was seen in children with bronchiolitis.

### Medications – antibiotics

Seventeen RCTs evaluated antibiotic-based interventions; nine on severe pneumonia, five on pneumonia, two RCTs on bronchiolitis, and one on SARI generally.

#### Antibiotics for severe pneumonia

Four RCTs evaluated oral amoxicillin vs parenteral antibiotics for severe pneumonia [26-29]. Three found oral amoxicillin vs injectable penicillin or ampicillin to have equal efficacy for children 3-59 months of age [26,27,29]. In a sub-analysis of a trial conducted by Addo Yobo et al., Jeena et al. [28] found greater treatment failure with oral amoxicillin or parenteral penicillin at day 2 and day 14 among children with human immunodeficiency virus (HIV) compared with children without HIV.

Five RCTs evaluated various parenteral antibiotic regimens, four of which included chloramphenicol [30-34]. Asghar et al. [30] found greater treatment failure with chloramphenicol compared to ampicillin/gentamicin in children aged 2-59 months across seven LMICs. No difference in cure rates was found with ceftriaxone vs penicillin plus chloramphenicol [31], mortality rates with benzylpenicillin plus chloramphenicol vs chloramphenicol alone [32], or mortality rates and adverse outcomes in penicillin/gentamicin vs chloramphenicol [33]. Ribeiro et al. [34] (Brazil) found hospital LOS and time to improvement of tachypnoea was shorter with oxacillin/ceftriaxone vs amoxicillin/clavulanic acid in hospitalised children diagnosed with very severe pneumonia.

#### Antibiotics for non-severe pneumonia

Four RCTs evaluated various antibiotic regimens for non-severe pneumonia [35-38]. Straus et al. [35] (Pakistan) found more treatment failures with trimethoprim-sulfamethoxazole (TMP-SMX) vs amoxicillin. Mulholland et al. [36] (The Gambia) found no difference in treatment failure with chloramphenicol vs TMP-SMX for the treatment of malnourished children with community-acquired pneumonia. Hasali et al. [37] (Malaysia) found shorter hospital LOS and time to switching to oral antibiotics with ampicillin-gentamicin combination compared to ampicillin alone. Brekhna et al. [38] (Pakistan) found no difference in clinical response as defined as oxygen requirement, feeding ability, or tachypnoea treated with amoxicillin vs cefuroxime vs clarithromycin, although this study was deemed to have a high risk of bias.

#### Antibiotics for bronchiolitis

Kabir et al. (Bangladesh) [39] found no benefit for parenteral ampicillin vs oral erythromycin vs no antibiotics on recovery of clinical symptoms in children with bronchiolitis; furthermore, children in the group given no antibiotics had a shorter hospital LOS compared with those who received oral antibiotics (mean 3.7 days vs 4.4 days). Pinto et al. [40] (Brazil) found that azithromycin had no effect on the duration of hospitalisation or oxygen requirement in children with bronchiolitis <1 year.

#### Antibiotics for undifferentiated SARI

Gamiño-Arroyo et al. [41] evaluated the effectiveness of nitazoxanide for SARI in patients over 12 months of age in Mexico and found no difference in hospital LOS and no difference in viral shedding.

### Medications – nebulised treatments

Seventeen studies evaluated nebulised treatments, including 16 on bronchiolitis and one on croup.

#### Nebulised treatments for bronchiolitis

Six studies evaluated different concentrations of nebulised saline for children with bronchiolitis. Five RCTs evaluated hypertonic (3%) vs (0.9%) normal saline (NS); three found no benefit for 3% saline on clinical status or hospital LOS [42-44]. Two RCTs found benefit for 3% saline. However, Ejaz et al. [45] (Pakistan) found greater reduction in respiratory score in the 3% saline group while Kumar et al. [46] (India) found greater improvement in clinical severity, oxygen saturation at 24 hours, and shorter hospital length of stay with 3% saline. Soleimani et al. [47] (Iran) compared three different concentrations of nebulised saline combined with salbutamol and found that, in children 1-24 months, those treated with 3% saline had shorter hospital LOS compared to those treated with 5% or NS.

Two studies evaluated nebulised salbutamol vs epinephrine, with mixed results; Ray et al. [48] (India) found lower clinical severity score and respiratory rate for salbutamol over epinephrine, while Adhikari et al. [49] (Nepal) found no difference in respiratory distress assessment instrument (RDAI) scores between those treated with epinephrine vs salbutamol. Three studies evaluated bronchodilators vs nebulised saline, two of which found benefit for bronchodilators; Jawaria et al. [50] (Pakistan) found that more children treated with salbutamol had a reduction in clinical bronchiolitis severity score compared to NS, although the study had high risk of bias; Khashabi et al. [51] (Iran) found improved oxygen saturation, clinical score, and respiratory rate in children treated with nebulised salbutamol or epinephrine compared to NS; however, Tinsa et al. [44] (Tunisia) found no difference in RDAI score or hospital LOS for nebulised terbutaline over saline. Shankar et al. [52] (India) found no significant difference in clinical severity scores or hospital LOS for nebulised hypertonic saline vs epinephrine in India. Gadomski et al. [53] (Egypt) compared nebulised albuterol, nebulised saline, orally administered albuterol, and orally administered placebo in Egypt and found no difference among the four groups, except for an improvement in respiratory rate among those with history of recurrent wheezing. Naz et al. [54] (Pakistan) found no difference in clinical severity score with nebulised NAC vs salbutamol. Pukai et al. [55] (Papua New Guinea) found that children <24 months with either bronchiolitis or pneumonia treated with NS vs standard of care had a greater reduction in respiratory distress score (RDS), and increase in pulse oximetry at four hours, and ability to be discharged from the ED.

#### Nebulised treatments for croup

Eghbali et al. [56] (Iran) found a reduction in Westley clinical croup scores for nebulised epinephrine plus dexamethasone vs nebulised saline plus dexamethasone in children aged six months to six years at 30, 60, and 90 minutes, although this difference was not seen at 120 minutes.

### Medications – nutrients and minerals

Seventeen studies evaluated nutrients and minerals (Vitamin A, C/E, D, and Zinc) as an intervention for treatment of children with acute lower respiratory tract infections (ALRTI), including pneumonia and bronchiolitis.

#### Vitamin A for ALRTI, including pneumonia

Three RCTs evaluated a single high dose of vitamin A among hospitalised children with ALRTIs. Donnen et al. [57] (Democratic Republic of the Congo) found no effect on mortality rates of a high-dose vitamin A vs low dose daily vitamin A on children with ALRTIs or diarrhoea. Similarly, Kiolhede et al. [58] (Guatemala) found no effect of adjuvant high dose vitamin A vs placebo on clinical parameters (e.g. respiratory rate, oxygen saturation), hospital LOS, or death. However, Julien et al. [59] (Mozambique) found a shorter median hospital LOS (four days vs three days) among children treated with single high-dose vitamin A.

Six RCTs evaluated vitamin A for children with pneumonia [60-65]. Four evaluated high-dose vitamin A given on the day of admission and an additional dose on the subsequent day, three of which (conducted in Tanzania, Peru, and Brazil) found no effect on mortality, hospital LOS, or duration of pneumonia symptoms [61,63,65]. Furthermore, Stephensen et al. [65] (Peru) additionally found greater adverse effects including lower oxygen saturation in children treated with high-dose vitamin A with pneumonia in Peru. However, Si et al. [60] (Vietnam) found no overall effect on hospital LOS in children with moderate-to-severe pneumonia treated with high-dose vitamin A, but a shorter hospital LOS among malnourished children (particularly females with very severe pneumonia) treated with high-dose vitamin A. Rodriguez et al. [64] found no difference with treatment with moderate-dose vitamin A on duration of pneumonia in children with non-measles pneumonia in Ecuador. Lastly, Hussey et al. [62] focused on patients with measles pneumonia and found lower mortality, shorter hospital LOS, and faster recovery in children who received vitamin A in South Africa.

#### Vitamin C and E for ALRTI, including pneumonia

Mahalanabis et al. [66] (India) evaluated Vitamin C and E together as adjunctive treatment for pneumonia in India and found no significant benefits of the combination therapy compared to placebo on tachypnoea, fever, feeding, or clinical status.

#### Vitamin D for ALRTI, including pneumonia

Six RCTs evaluated the effect of vitamin D in children under five years with ALRTI or pneumonia, all of which found no benefit for vitamin D [67-72]. Somnath et al. [67] (India), Choudhary et al. [68] (India), Gupta et al. [69] (India), Dhungel et al. [71] (Pakistan), and Manaseki-Holland et al [72] (Afghanistan) found no effect of a single high dose of vitamin D on hospital LOS or mortality in children under five years hospitalised with ALRTI. Similarly, Rajshekhar et al. [70] (India) found no difference in time to resolution of pneumonia symptoms.

#### Zinc for bronchiolitis

Two RCTs evaluated the effect of zinc on bronchiolitis in children under two years [73,74]. Ahadi et al. [73] found that children in the treatment group had shorter mean hospital LOS (4.14 (standard deviation (SD) = 1.21) vs 4.64 (SD = 1.2) days; *P* = 0.016) and less wheezing and rhinorrhoea at 72 hours (16% vs 36%, *P* = 0.023, 0 vs 12%, *P* = 0.027, respectively). Both found no difference in tachypnoea, retractions, or cyanosis at 24 hours in the treatment vs control group; both studies were deemed to have high risk of bias.

#### Zinc for pneumonia

The effect of zinc on pneumonia outcomes were evaluated in 15 RCTs [75-89]. Seven studies found no benefit for zinc treatment over placebo [75,79,81,82,85,86,89]. Five studies reported favourable outcomes for zinc; Brooks et al. [76] (Bangladesh) found a shorter duration to resolution of pneumonia and a one-day shorter hospital LOS in the zinc group (72 vs 96 hours; hazard ratio (HR) = 0.7; 95% CI = 0.51-0.98), but no differences in resolution of chest indrawing or hypoxia; Heydarian et al. [80] (Iran) found no benefit for zinc on hospital LOS but found reported shorter duration of fever at 24 and 36 hours and improvement in tachypnoea at 36 hours; Qasemzadeh et al. [83] (Iran) and Rerksuppaphol et al. [84] (Thailand) found shorter hospital LOS and time to resolution of clinical symptoms; Srinivasan et al. [87] (Uganda) found decreased case fatality rates in children with severe pneumonia treated with zinc (4.0% vs 11.9%), with greater effects in children with HIV. Conversely, Coles et al. [77] (India) reported an increase in the median hospital LOS in the zinc group, but the study had high risk of bias. Howie et al. [78] (The Gambia) reported mixed outcomes with no difference in time to resolution of symptoms, but marginal benefit for reduced time to resolution of chest indrawing and sternal retraction with zinc. Wadhwa et al. [88] (India) found no benefit for zinc in their overall study population with severe pneumonia, but found benefit following a stratified analysis among those with very severe pneumonia.

### Medications – steroids

Nine studies evaluated steroids; six on bronchiolitis, two on PJP pneumonia, and one on croup.

#### Steroids for bronchiolitis

Six RCTs evaluated steroids for bronchiolitis, four of which found no benefit for steroids on hospital LOS, duration of symptoms or Respiratory Distress Assessment Instrument (RDAI) score [90-93]. However, Teeratakulpisarn et al. [94] (Thailand) found decreased time to resolution of respiratory distress, duration of symptoms, duration of oxygen therapy, and hospital LOS in children treated with single IM dexamethasone, and Virk et al. [91] (Pakistan) found decreased rhonchi on day three of admission and shorter hospital LOS with oral prednisolone; however, this this study was found to have a high risk of bias.

#### Steroids for croup, PJP pneumonia

Sumboonnanonda et al. [95] (Thailand) found children treated with parenteral dexamethasone treatment had lower croup scores at 48 hours, shorter LOS and lower incidence of endotracheal intubation compared to placebo. Green et al. [96] (South Africa) and Newberry et al. [109] (Malawi) found that children with HIV and PJP pneumonia treated with adjunctive steroids had lower mortality rates.

#### Medications – other

Dawood et al. [97] found no effect for oseltamivir in children with influenza on LOS or work of breathing in Panama. Yohannes et al. [98] (Indonesia) and Becina Paolo Gene et al. [99] (Philippines) found no benefit for adjuvant probiotics in children with pneumonia. Shang et al. [100] (China) found children with bronchiolitis treated with *laggera pterodonta* (traditional Chinese medicine herb), more frequently met discharge criteria at 96 and 120 hours compared to control group.

## DISCUSSION

Paediatric SARIs constitute a large burden of disease globally, with children in LMICs having the highest risk of mortality. Given the association between delay of acute interventions and poor outcomes, we examined the current evidence on which emergency care interventions (those delivered in early period of care) have the strongest evidence for benefit on clinical outcomes. Patients generally present to a clinical provider without a specific disease or microbiologic diagnosis. Thus, our use of the SARI criteria as screening criteria was meant to capture all studies including patients with undifferentiated respiratory illness. This strategy resulted in a broad array of interventions being included in the review, with most studies focused on medication interventions for children with pneumonia/ALRTIs or bronchiolitis (the most common respiratory conditions leading to hospitalisation in children globally). Few interventions had strong evidence for benefit, the strongest being for respiratory support interventions. There was also strong evidence for a lack of benefit of early use of adjuvant treatments such as Vitamin A, D, and zinc on key clinical outcomes such as mortality and hospital length-of-stay for pneumonia and bronchiolitis.

### Respiratory support interventions, including bCPAP

Strong oxygen delivery systems decrease risk of death, although results were inconclusive for the utility of CPAP. The included studies showed that respiratory support interventions should be implemented early, regardless of the final diagnosis: pneumonia, bronchiolitis, croup, other disease, or undifferentiated. Particularly, interventions of equipment and supplies to deliver oxygen – concentrators, nasal devices, and masks, must be readily available and combined with standardised training, technical support for maintenance of devices, pulse oximetry to monitor the patient, and guidelines for escalation and weaning of oxygen therapy. As a package, these interventions were found to decrease risk of death of a child with pneumonia by more than one third.

CPAP was found to be very beneficial in children <1 year of age and somewhat beneficial in children <5 years of age with pneumonia in LMICs. However, this intervention should be used cautiously as it can be particularly cumbersome for small children and requires extra financial, material, and human resources to maintain. A 2021 systematic review evaluated CPAP for children with respiratory distress in resource-limited settings and found the existing evidence is inconclusive for CPAP efficacy against death and adverse events, compared with oxygen [101]. The authors also pointed out the variations in resource availability, level of care available (ICU vs ward), and illness severity make the context in which interventions were assessed important for extrapolating results.

### Steroids and other treatments for bronchiolitis

Based on our findings, steroids were not shown to be beneficial for bronchiolitis, but are beneficial for patients with HIV and PJP pneumonia, confirming the findings from earlier studies, including several systematic reviews finding a lack of convincing evidence for β2 agonists and anticholinergics, adrenaline, corticosteroids, hypertonic saline, antibiotics, and chest physiotherapy in the acute management of bronchiolitis, as reflected in current clinical practice guidelines [102].

International treatment guidelines do not recommend nebulised saline for bronchiolitis, though a recent Cochrane review of articles from high- and low-income countries reported potentially improved outcomes (hospital LOS, clinical severity) based on low to moderate quality evidence [103]. In our review, one study with low risk of bias indicated nebulised NS may be beneficial for children with bronchiolitis [55]. However, the remaining eight studies that evaluated nebulised saline (either normal saline or hypertonic) with various comparators showed either no significant clinical outcome improvement or had a high risk of bias.

We found studies with mixed findings on nebulised salbutamol for bronchiolitis [48,49], but with supportive results when comparing benefit of nebulised epinephrine for croup [56]. Though some had possible biases, this intervention should be considered as a targeted question for future international guideline updates.

### Early administration of adjuvant nutrients and minerals

Vitamin A only proved to be beneficial for children with measles and vitamin D only helpful for preventing repeat pneumonia [62,72]. A dedicated review of vitamin D interventions for ALRTI international guideline development could be considered. Vitamin A, C, or D use in other aetiologies of SARI were not supported by the evidence, whether due to negative studies or risk of bias.

The two studies that evaluated zinc for bronchiolitis both had high risk of bias associated with them. While Farhad et al. [74] found zinc to provide some clinical improvement, this has not been replicated in other studies, and zinc is not a recommended intervention for bronchiolitis by WHO guidelines [104]. However, among the sixteen studies that evaluated zinc for pneumonia, only one had high risk of bias associated. While most studies did not report significant clinical improvement in patients with pneumonia receiving zinc, and it is not a recommended standard intervention directly for pneumonia, zinc is an important micronutrient for a child’s overall health and development and is often deficient in children who are dehydrated [105,106]. It is possible that the children enrolled in studies that found that zinc had an effect on the improvement of clinical outcomes were more dehydrated than in other studies. Zinc remains an important acute care intervention for children who are deficient or at risk of dehydration, but not part of regular home or inpatient treatment of children with pneumonia.

### Antibiotics

Antibiotics for pneumonia continue to be a recommended treatment according to international guidelines [104,107]. Differentiation is made between diagnoses and treatments for pneumonia and severe pneumonia. Pneumonia is a form of ALRTI and treated with oral amoxicillin among outpatient children, unless they have a HIV infection. Severe pneumonia is recommended to be treated initially with intravenous (IV) or intramuscular (IM) ampicillin (or benzylpenicillin) and gentamicin, switching to IV/IM gentamicin and cloxacillin if no improvement is observed in 48 hours and ceftriaxone in the case of failure of first-line treatment [104,107,108]. All three studies evaluating antibiotic regimens for severe pneumonia in children without underlying complications found treatment with high-dose oral amoxicillin equivalent to IV/IM ampicillin and recommended an update to current WHO guidelines. All were deemed to have some risk of bias, but similar findings were replicated in nine countries across the three studies. We agree that this recommendation should be re-evaluated in international guidelines. Replacing parenteral antibiotics with an oral regimen for severe pneumonia would save time and money from hospitalisation, including risk reduction of nosocomial infections. Ribeiro et al. [34] found a significant improvement in tachypnoea and decreased length of hospital stay in the amoxicillin group in comparing IV oxacillin plus ceftriaxone vs amoxicillin plus clavulanic acid for children (initiated IV and changed to oral after 48 hours) with very severe pneumonia.

Based on our findings, chloramphenicol is not a viable option for severe pneumonia treatment and is not generally included as part of usual care for this illness [104]. Studies evaluating different antibiotic regimens for non-severe pneumonia found that cotrimoxazole is not a reliable first-line choice, and gentamicin is not required as a first line for community acquired pneumonia. Other regimens did not reveal a superior choice.

Multiple studies confirmed a lack of benefit antibiotics for treatment of viral-mediated illness (including bronchiolitis) supporting recommendations that antibiotics should not be used for viral respiratory infection. Gamino-Arroyo’s [41] evaluation of nitazoxanide in addition to standard of care in children >1 year of age in Mexico for influenza-like illness, and Kabir et al. [39] and Pinto et al.’s [40] evaluation of antibiotics for viral bronchiolitis are important additions to the literature supporting a lack of effectiveness of antibiotics against viral illnesses, both in terms of lack of symptom improvement and in viral dynamics.

### Critical gaps in the literature

Very few studies included populations with undifferentiated respiratory distress, and only one study used the case definition of SARI as its inclusion criteria. Given that EC interventions often must be implemented prior to a formal clinical diagnosis, our findings highlight a need for future studies to enrol children with undifferentiated respiratory infections. Most of the included studies (>80%) evaluated medication interventions, with relatively few focusing on other interventions such as respiratory support. We also found that very few studies explicitly reported on the timing of interventions, with most studies either conducted outside of dedicated emergency wards or clearly during the earliest periods of care when the patient’s respiratory illness may be most amenable to acute interventions (e.g. shortly after presentation to a health facility, such as in triage or a dedicated emergency unit).

This review has several limitations. Those related to the development of the review and overall characteristics of results are described elsewhere [7]. Given that emergency care is not well defined globally (especially in LMICs) and emergency care is often delivered outside of a dedicated emergency unit, we agreed on including any “emergent and early intervention” deemed appropriate by the reviewers, specifically those focused on community-acquired pneumonia or discussing interventions beginning in the first 24 hours of hospitalisation. As timing of interventions may greatly impact the trajectory of disease progression, future research on early and emergency care of patients must report timing and location of intervention in publications. Furthermore, inclusion of specific elements in the search strategy such as “bronchiolitis”, “croup” or “children” may have supported a smaller search result and tailored review.

## CONCLUSION

Despite few studies specifically evaluating patients in dedicated emergency units, or those with undifferentiated SARIs, a wide variety of interventions that may impact the early care of children was found. While the burden of SARI in paediatric populations is high, few interventions had high quality evidence for benefit on clinical outcomes in LMICs, with respiratory support interventions having the strongest evidence for decreasing symptom duration and hospital length of stay, but with inconclusive evidence for bubble CPAP. Despite relatively few studies focused on non-medication interventions, the strongest evidence of benefit was found for respiratory support interventions such as improved oxygen delivery systems to decrease risk of death. Mixed results were found for interventions for bronchiolitis, although possible benefits were found for hypertonic nebulised saline to decrease hospital length of stay. Early use of adjuvant treatments such as Vitamin A, D and zinc did not appear to have convincing evidence of benefit on clinical outcomes in pneumonia and bronchiolitis. Further research evaluating the effect of targeted and time sensitive interventions for children with undifferentiated severe respiratory infections is greatly needed.

## Additional material


Online Supplementary Document
