# Supplementary material for: Emergency care interventions for paediatric severe acute respiratory infections in low- and middle-income countries: A systematic review and narrative synthesis
Source: J Glob Health. 2023 Jun 9;13:04065. doi: 10.7189/jogh.13.04065 (PMC10248949; doi:10.7189/jogh.13.04065)

ONLINE SUPPLEMENTARY DOCUMENT

Title: Emergency care interventions for pediatric severe acute respiratory infections in low- and middle-income countries: a systematic review and narrative synthesis

Authors:

Pryanka Relan MD MPH
Stephanie Chow Garbern MD MPH
Gerard M O'Reilly MBBS FACEM MPH MBiostat AStat PhD
Corey B Bills MD MPH
Megan Schultz MD MA
Indi Trehan MD MPH DTM&H
Sean M Kivlehan MD MPH
Torben K Becker MD PhD

Appendix S1
Search strategy

PubMed, Global Health, and Global Index Medicus databases were searched using a combination of controlled vocabulary and text word search terms, combining the concepts of severe acute respiratory infections, emergency care interventions, and low/middle-income countries as defined by World Bank Classification. Searches were conducted on November 30 2020 and January 21 2021. The results were exported to EndNote 20, and deduplicated prior to uploading the resulting reference set to Covidence for screening.

Search Strategy:

**Name of Search**	Impact of Emergency Care Interventions on Severe Acute Respiratory Infection Outcomes in Low- and Middle-Income Countries
**Databases Searched**	PubMed/MEDLINE, Global Health, Global Index Medicus
**Inclusion Criteria**	*Population*: Patients with severe acute respiratory infections in LMICs (World Bank Classification), pediatric and adult<br>*Intervention*: Emergency care interventions (medications, respiratory support, supportive care, triage, monitoring)<br>*Study type*: RCTs, observational studies with control group, observational studies with pre/post design<br>*Language*: English, Spanish
Exclusion Criteria	Population: Patients in high income countries, neonates, patients with chronic respiratory infections, patients with mild respiratory infections not requiring hospitalization, patients with sepsis or ARDS not due to a respiratory infection.<br>Intervention: Educational interventions, diagnostic tools, chronic disease management, preventative care, severity scores, prognostic scores<br>Study type: Case reports, case series, opinion papers, descriptive papers, abstracts
Concept 1	Severe acute respiratory infections (SARI)
Concept 2	Emergency care interventions
Concept 3	RCTs, observational studies with control group or pre/post design
Concept 4	Low and middle income countries

Database	PubMed/MEDLINE
Platform	PubMed
Years Searched	1940s – Present
Date Last Searched	November 30, 2020
Limits	None
Number of Results	17,286
Notes	

#1	("acute respiratory distress syndrome"[Text Word] OR "adenoviridae infections"[MeSH Terms] OR "bronchiolitis"[Text Word] OR "bronchitis"[Text Word] OR "bronchopneumonia"[Text Word] OR "coronavirus infections"[MeSH Terms] OR "coronavirus"[Text Word] OR "covid 19"[Supplementary Concept] OR "covid*"[Text Word] OR "croup"[Text Word] OR "epiglottitis"[Text Word] OR "flu"[Text Word] OR "h1n1"[Text Word] OR "influenza"[Text Word] OR "laryngitis"[Text Word] OR "laryngotracheobronchitis"[Text Word] OR "mers virus"[Text Word] OR "mers cov"[Text Word] OR "middle east respiratory syndrome"[Text Word] OR "ncov*"[Text Word] OR "orthomyxoviridae infections"[MeSH Terms] OR "picornaviridae infections"[MeSH Terms] OR "pleurisy"[Text Word] OR "pleuropneumonia"[Text Word] OR "pneumonia"[Text Word] OR "pneumovirus infections"[MeSH Terms] OR "respiratory depression"[Text Word] OR "respiratory failure"[Text Word] OR "respiratory infection*"[Text Word] OR ("respiratory insufficiency"[MeSH Terms] OR "pulmonary valve insufficiency"[MeSH Terms]) OR "respiratory insufficiency"[Text Word] OR "respiratory syncytial virus infections"[MeSH Terms] OR "respiratory syncytial virus"[Text Word] OR "respiratory tract infection*"[Text Word] OR "respiratory tract infections"[MeSH Terms] OR "respiratory viral infections"[Text Word] OR "rhinitis"[Text Word] OR "rsv"[Text Word] OR "sars cov2"[Text Word] OR "severe acute respiratory infection*"[Text Word] OR "severe acute respiratory syndrome coronavirus 2"[Supplementary Concept] OR "tuburculosis"[Text Word] OR "whooping cough"[Text Word] OR "mycoplasma pneumoniae"[Text Word] OR "pleural effusion"[Text Word] OR "pertussis"[Text Word] OR "parapneumonic effusion"[Text Word] OR "hypoxia"[Text Word] OR "hypoxemia"[Text Word] OR "respiratory distress"[Text Word] OR "pulmonary abscess"[Text Word] OR "wheezing"[Text Word] OR "stridor"[Text Word] OR "crackles"[Text Word] OR "rhonchi"[Text Word] OR "rales"[Text Word] OR "empyema"[Text Word] OR "pyothorax"[Text Word])
#2	("levalbuterol"[MeSH Terms] OR "albuterol"[MeSH Terms] OR "albuterol"[Text Word] OR "anti biotic"[Text Word] OR "anti infective agents"[MeSH Terms] OR "anti infective"[Text Word] OR "anti viral"[Text Word] OR "antibiotic"[Text Word] OR "antiviral agents"[MeSH Terms] OR "antiviral agents"[Supplementary Concept] OR "antiviral"[Text Word] OR "artificial respiration"[Text Word] OR "atomizer"[Text Word] OR "continuous monitoring"[Text Word] OR "continuous positive airway pressure"[Text Word] OR "corticosteroids"[Text Word] OR "cpap"[Text Word] OR "drug"[Text Word] OR "face down"[Text Word] OR "face down"[Text Word] OR "inhaler"[Text Word] OR "intubation, intratracheal"[MeSH Terms] OR "mechanical ventilation"[Text Word] OR "medication"[Text Word] OR "micronutrients"[MeSH Terms] OR "monitoring, physiologic"[MeSH Terms] OR "nebulizer"[Text Word] OR "nebulizers and vaporizers"[MeSH Terms] OR "non invasive ventilation"[Text Word] OR "noninvasive ventilation"[Text Word] OR "oximetry"[MeSH Terms] OR "oxygen/therapeutic use"[MeSH Terms] OR "point of care systems"[MeSH Terms] OR "position*"[Text Word] OR "postur*"[Text Word] OR "posture"[MeSH Terms] OR "prone"[Text Word] OR "pulse oximetry"[Text Word] OR "respiratory support"[Text Word] OR "respiratory system agents"[MeSH Terms] OR "respiratory therapy"[MeSH Terms] OR "risk stratification"[Text Word] OR "risk stratify"[Text Word] OR "side lying"[Text Word] OR "side lying"[Text Word] OR "side lying"[Text Word] OR "steroid"[Text Word] OR "supine"[Text Word] OR "therapeutic"[Text Word] OR "therapeutics"[MeSH Terms] OR "treat"[Text Word] OR "treatment"[Text Word] OR "upright"[Text Word] OR "vaporizer"[Text Word] OR "zinc"[Text Word] OR "vitamin"[Text Word] OR "nasal cannula"[Text Word] OR "oxygen

	therapy"[Text Word] OR "continuous positive airway pressure"[MeSH Terms] OR "oxygen inhalation therapy"[MeSH Terms] OR "adjuvant"[Text Word] OR "supportive care"[Text Word] OR "prehospital"[Text Word] OR "pre hospital"[Text Word] OR "salbutamol"[Text Word] OR "blow by"[Text Word] OR "face mask"[Text Word] OR "oxygen tent"[Text Word] OR "bag valve mask"[Text Word] OR "bag valve mask"[Text Word] OR "supplemental oxygen"[Text Word] OR "acute care"[Text Word] OR "critical care"[MeSH Terms] OR "critical care"[Text Word] OR "emergencies"[MeSH Terms] OR "emergencies"[Text Word] OR "emergency medical services"[MeSH Terms] OR "emergency medicine"[MeSH Terms] OR "emergency treatment"[MeSH Terms] OR "emergency"[Text Word] OR "emergicenters"[Text Word] OR "evidence based emergency medicine"[MeSH Terms] OR "triage"[Text Word])
#3	"case control"[Title/Abstract] OR "case comparison"[Title/Abstract] OR "case controlled"[Title/Abstract] OR "case referent"[Title/Abstract] OR "case comparison"[Title/Abstract] OR "case control studies"[MeSH Terms] OR "controlled clinical trial"[Publication Type] OR "epidemiologic studies"[MeSH Terms] OR "observational study"[Publication Type] OR "placebo"[Title/Abstract] OR "randomised"[Text Word] OR "randomized controlled trial"[Publication Type] OR "randomized"[Title/Abstract] OR "retrospective"[Title/Abstract] OR "randomized controlled trials as topic"[MeSH Terms] OR "multicenter study"[Publication Type] OR "multicenter studies as topic"[MeSH Terms] OR "observational studies as topic"[MeSH Terms]
#4	("developing countries"[MeSH Terms] OR "africa"[MeSH Terms] OR "asia"[MeSH Terms:noexp] OR "asia, central"[MeSH Terms] OR "asia, southeastern"[MeSH Terms] OR "asia, western"[MeSH Terms] OR "caribbean region"[MeSH Terms] OR "south america"[MeSH Terms] OR "latin america"[MeSH Terms] OR "central america"[MeSH Terms] OR "afghanistan"[MeSH Terms] OR "albania"[MeSH Terms] OR "algeria"[MeSH Terms] OR "american samoa"[MeSH Terms] OR "angola"[MeSH Terms] OR "antigua and barbuda"[MeSH Terms] OR "argentina"[MeSH Terms] OR "armenia"[MeSH Terms] OR "azerbaijan"[MeSH Terms] OR "bahrain"[MeSH Terms] OR "bangladesh"[MeSH Terms] OR "barbados"[MeSH Terms] OR "benin"[MeSH Terms] OR "republic of belarus"[MeSH Terms] OR "belize"[MeSH Terms] OR "bhutan"[MeSH Terms] OR "bolivia"[MeSH Terms] OR "bosnia and herzegovina"[MeSH Terms] OR "botswana"[MeSH Terms] OR "brazil"[MeSH Terms] OR "bulgaria"[MeSH Terms] OR "burkina faso"[MeSH Terms] OR "burundi"[MeSH Terms] OR "cambodia"[MeSH Terms] OR "cameroon"[MeSH Terms] OR "cabo verde"[MeSH Terms] OR "central african republic"[MeSH Terms] OR "chad"[MeSH Terms] OR "chile"[MeSH Terms] OR "china"[MeSH Terms] OR "colombia"[MeSH Terms] OR "comoros"[MeSH Terms] OR "congo"[MeSH Terms] OR "costa rica"[MeSH Terms] OR "cote d ivoire"[MeSH Terms] OR "croatia"[MeSH Terms] OR "cuba"[MeSH Terms] OR "cyprus"[MeSH Terms] OR "czechoslovakia"[MeSH Terms] OR "czech republic"[MeSH Terms] OR "slovakia"[MeSH Terms] OR "djibouti"[MeSH Terms] OR "democratic republic of the congo"[MeSH Terms] OR "dominica"[MeSH Terms] OR "dominican republic"[MeSH Terms] OR "timor leste"[MeSH Terms] OR "ecuador"[MeSH Terms] OR "egypt"[MeSH Terms] OR "el salvador"[MeSH Terms] OR "eritrea"[MeSH Terms] OR "estonia"[MeSH Terms] OR "ethiopia"[MeSH Terms] OR "fiji"[MeSH Terms] OR "gabon"[MeSH Terms] OR "gambia"[MeSH Terms] OR "georgia republic"[MeSH Terms] OR "ghana"[MeSH Terms] OR "greece"[MeSH Terms] OR "grenada"[MeSH Terms] OR "guatemala"[MeSH Terms] OR "guinea"[MeSH Terms] OR "guinea bissau"[MeSH Terms] OR "guam"[MeSH Terms] OR "guyana"[MeSH Terms] OR "haiti"[MeSH Terms] OR "honduras"[MeSH Terms] OR "hungary"[MeSH Terms] OR "india"[MeSH Terms] OR

"indonesia"[MeSH Terms] OR "iran"[MeSH Terms] OR "iraq"[MeSH Terms] OR "jamaica"[MeSH Terms] OR "jordan"[MeSH Terms] OR "kazakhstan"[MeSH Terms] OR "kenya"[MeSH Terms] OR "korea"[MeSH Terms] OR "kosovo"[MeSH Terms] OR "kyrgyzstan"[MeSH Terms] OR "laos"[MeSH Terms] OR "latvia"[MeSH Terms] OR "lebanon"[MeSH Terms] OR "lesotho"[MeSH Terms] OR "liberia"[MeSH Terms] OR "libya"[MeSH Terms] OR "lithuania"[MeSH Terms] OR "republic of north macedonia"[MeSH Terms] OR "madagascar"[MeSH Terms] OR "malaysia"[MeSH Terms] OR "malawi"[MeSH Terms] OR "mali"[MeSH Terms] OR "malta"[MeSH Terms] OR "mauritania"[MeSH Terms] OR "mauritius"[MeSH Terms] OR "mexico"[MeSH Terms] OR "micronesia"[MeSH Terms] OR "middle east"[MeSH Terms] OR "moldova"[MeSH Terms] OR "mongolia"[MeSH Terms] OR "montenegro"[MeSH Terms] OR "morocco"[MeSH Terms] OR "mozambique"[MeSH Terms] OR "myanmar"[MeSH Terms] OR "namibia"[MeSH Terms] OR "nepal"[MeSH Terms] OR "netherlands antilles"[MeSH Terms] OR "new caledonia"[MeSH Terms] OR "nicaragua"[MeSH Terms] OR "niger"[MeSH Terms] OR "nigeria"[MeSH Terms] OR "oman"[MeSH Terms] OR "pakistan"[MeSH Terms] OR "palau"[MeSH Terms] OR "panama"[MeSH Terms] OR "papua new guinea"[MeSH Terms] OR "paraguay"[MeSH Terms] OR "peru"[MeSH Terms] OR "philippines"[MeSH Terms] OR "poland"[MeSH Terms] OR "portugal"[MeSH Terms] OR "puerto rico"[MeSH Terms] OR "romania"[MeSH Terms] OR "russia"[MeSH Terms] OR "russia pre 1917"[MeSH Terms] OR "rwanda"[MeSH Terms] OR "saint kitts and nevis"[MeSH Terms] OR "saint lucia"[MeSH Terms] OR "saint vincent and the grenadines"[MeSH Terms] OR "samoa"[MeSH Terms] OR "saudi arabia"[MeSH Terms] OR "senegal"[MeSH Terms] OR "serbia"[MeSH Terms] OR "montenegro"[MeSH Terms] OR "seychelles"[MeSH Terms] OR "sierra leone"[MeSH Terms] OR "slovenia"[MeSH Terms] OR "sri lanka"[MeSH Terms] OR "somalia"[MeSH Terms] OR "south africa"[MeSH Terms] OR "sudan"[MeSH Terms] OR "suriname"[MeSH Terms] OR "eswatini"[MeSH Terms] OR "syria"[MeSH Terms] OR "tajikistan"[MeSH Terms] OR "tanzania"[MeSH Terms] OR "thailand"[MeSH Terms] OR "togo"[MeSH Terms] OR "tonga"[MeSH Terms] OR "trinidad and tobago"[MeSH Terms] OR "tunisia"[MeSH Terms] OR "turkey"[MeSH Terms] OR "turkmenistan"[MeSH Terms] OR "uganda"[MeSH Terms] OR "ukraine"[MeSH Terms] OR "uruguay"[MeSH Terms] OR "ussr"[MeSH Terms] OR "uzbekistan"[MeSH Terms] OR "vanuatu"[MeSH Terms] OR "venezuela"[MeSH Terms] OR "vietnam"[MeSH Terms] OR "yemen"[MeSH Terms] OR "yugoslavia"[MeSH Terms] OR "zambia"[MeSH Terms] OR "zimbabwe"[MeSH Terms] OR "developing country"[Text Word] OR "developing countries"[Text Word] OR "developing nation"[Text Word] OR "developing nations"[Text Word] OR "developing population"[Text Word] OR "developing populations"[Text Word] OR "developing world"[Text Word] OR "less developed country"[Text Word] OR "less developed countries"[Text Word] OR "less developed nation"[Text Word] OR "less developed nations"[Text Word] OR "less developed world"[Text Word] OR "lesser developed countries"[Text Word] OR "lesser developed nations"[Text Word] OR "least developed country"[Text Word] OR "least developed countries"[Text Word] OR "least developed nations"[Text Word] OR "under developed country"[Text Word] OR "under developed countries"[Text Word] OR "under developed nations"[Text Word] OR "under developed world"[Text Word] OR "underdeveloped country"[Text Word] OR "underdeveloped countries"[Text Word] OR "underdeveloped nation"[Text Word] OR "underdeveloped nations"[Text Word] OR "underdeveloped population"[Text Word] OR "underdeveloped populations"[Text Word] OR "underdeveloped world"[Text Word] OR "middle income country"[Text Word] OR "middle income countries"[Text Word] OR "middle income nation"[Text Word] OR "middle income nations"[Text Word] OR

"middle income population"[Text Word] OR "middle income populations"[Text Word] OR "low income country"[Text Word] OR "low income countries"[Text Word] OR "low income nation"[Text Word] OR "low income nations"[Text Word] OR "low income population"[Text Word] OR "low income populations"[Text Word] OR "lower income country"[Text Word] OR "lower income countries"[Text Word] OR "lower income nations"[Text Word] OR "lower income population"[Text Word] OR "lower income populations"[Text Word] OR "underserved countries"[Text Word] OR "underserved nations"[Text Word] OR "underserved population"[Text Word] OR "underserved populations"[Text Word] OR "under served population"[Text Word] OR "under served populations"[Text Word] OR "deprived countries"[Text Word] OR "deprived population"[Text Word] OR "deprived populations"[Text Word] OR "poor country"[Text Word] OR "poor countries"[Text Word] OR "poor nation"[Text Word] OR "poor nations"[Text Word] OR "poor population"[Text Word] OR "poor populations"[Text Word] OR "poor world"[Text Word] OR "poorer countries"[Text Word] OR "poorer nations"[Text Word] OR "poorer population"[Text Word] OR "poorer populations"[Text Word] OR "developing economy"[Text Word] OR "developing economies"[Text Word] OR "less developed economy"[Text Word] OR "less developed economies"[Text Word] OR "underdeveloped economies"[Text Word] OR "middle income economy"[Text Word] OR "middle income economies"[Text Word] OR "low income economy"[Text Word] OR "low income economies"[Text Word] OR "lower income economies"[Text Word] OR "low gdp"[Text Word] OR "low gnp"[Text Word] OR "low gross domestic"[Text Word] OR "low gross national"[Text Word] OR "lower gdp"[Text Word] OR "lower gross domestic"[Text Word] OR "lmic"[Text Word] OR "lmics"[Text Word] OR "third world"[Text Word] OR "lami country"[Text Word] OR "lami countries"[Text Word] OR "transitional country"[Text Word] OR "transitional countries"[Text Word] OR "africa"[Text Word] OR "asia"[Text Word] OR "west indies"[Text Word] OR "south america"[Text Word] OR "latin america"[Text Word] OR "central america"[Text Word] OR "afghanistan"[Text Word] OR "albania"[Text Word] OR "algeria"[Text Word] OR "angola"[Text Word] OR "antigua"[Text Word] OR "barbuda"[Text Word] OR "argentina"[Text Word] OR "armenia"[Text Word] OR "armenian"[Text Word] OR "aruba"[Text Word] OR "azerbaijan"[Text Word] OR "bahrain"[Text Word] OR "bangladesh"[Text Word] OR "barbados"[Text Word] OR "benin"[Text Word] OR "byelarus"[Text Word] OR "byelorussian"[Text Word] OR "belarus"[Text Word] OR "belorussian"[Text Word] OR "belorussia"[Text Word] OR "belize"[Text Word] OR "bhutan"[Text Word] OR "bolivia"[Text Word] OR "bosnia"[Text Word] OR "herzegovina"[Text Word] OR "hercegovina"[Text Word] OR "botswana"[Text Word] OR "brasil"[Text Word] OR "brazil"[Text Word] OR "bulgaria"[Text Word] OR "burkina faso"[Text Word] OR "burkina fasso"[Text Word] OR "upper volta"[Text Word] OR "burundi"[Text Word] OR "urundi"[Text Word] OR "cambodia"[Text Word] OR "khmer republic"[Text Word] OR "kampuchea"[Text Word] OR "cameroon"[Text Word] OR "cameroons"[Text Word] OR "cameron"[Text Word] OR "cape verde"[Text Word] OR "central african republic"[Text Word] OR "chad"[Text Word] OR "chile"[Text Word] OR "china"[Text Word] OR "colombia"[Text Word] OR "comoros"[Text Word] OR "comoro islands"[Text Word] OR "comores"[Text Word] OR "mayotte"[Text Word] OR "congo"[Text Word] OR "zaire"[Text Word] OR "costa rica"[Text Word] OR "cote d ivoire"[Text Word] OR "ivory coast"[Text Word] OR "croatia"[Text Word] OR "cuba"[Text Word] OR "cyprus"[Text Word] OR "czechoslovakia"[Text Word] OR "czech republic"[Text Word] OR "slovakia"[Text Word] OR "slovak republic"[Text Word] OR "djibouti"[Text Word] OR "french somaliland"[Text Word] OR "dominica"[Text Word] OR "dominican republic"[Text Word] OR "east timor"[Text Word] OR "timor leste"[Text Word] OR "ecuador"[Text Word]

OR "egypt"[Text Word] OR "united arab republic"[Text Word] OR "el salvador"[Text Word] OR "eritrea"[Text Word] OR "estonia"[Text Word] OR "ethiopia"[Text Word] OR "fiji"[Text Word] OR "gabon"[Text Word] OR "gabonese republic"[Text Word] OR "gambia"[Text Word] OR "gaza"[Text Word] OR "georgia republic"[Text Word] OR "georgian republic"[Text Word] OR "ghana"[Text Word] OR "gold coast"[Text Word] OR "greece"[Text Word] OR "grenada"[Text Word] OR "guatemala"[Text Word] OR "guinea"[Text Word] OR "guam"[Text Word] OR "guiana"[Text Word] OR "guyana"[Text Word] OR "haiti"[Text Word] OR "honduras"[Text Word] OR "hungary"[Text Word] OR "india"[Text Word] OR "maldives"[Text Word] OR "indonesia"[Text Word] OR "iran"[Text Word] OR "iraq"[Text Word] OR "isle of man"[Text Word] OR "jamaica"[Text Word] OR "jordan"[Text Word] OR "kazakhstan"[Text Word] OR "kazakh"[Text Word] OR "kenya"[Text Word] OR "kiribati"[Text Word] OR "korea"[Text Word] OR "kosovo"[Text Word] OR "kyrgyzstan"[Text Word] OR "kirghizia"[Text Word] OR "kyrgyz republic"[Text Word] OR "kirghiz"[Text Word] OR "kirgizstan"[Text Word] OR "lao pdr"[Text Word] OR "laos"[Text Word] OR "latvia"[Text Word] OR "lebanon"[Text Word] OR "lesotho"[Text Word] OR "basutoland"[Text Word] OR "liberia"[Text Word] OR "libya"[Text Word] OR "lithuania"[Text Word] OR "macedonia"[Text Word] OR "madagascar"[Text Word] OR "malagasy republic"[Text Word] OR "malaysia"[Text Word] OR "malaya"[Text Word] OR "malay"[Text Word] OR "sabah"[Text Word] OR "sarawak"[Text Word] OR "malawi"[Text Word] OR "nyasaland"[Text Word] OR "mali"[Text Word] OR "malta"[Text Word] OR "marshall islands"[Text Word] OR "mauritania"[Text Word] OR "mauritius"[Text Word] OR "agalega islands"[Text Word] OR "mexico"[Text Word] OR "micronesia"[Text Word] OR "moldova"[Text Word] OR "moldovia"[Text Word] OR "moldovian"[Text Word] OR "mongolia"[Text Word] OR "montenegro"[Text Word] OR "morocco"[Text Word] OR "ifni"[Text Word] OR "mozambique"[Text Word] OR "myanmar"[Text Word] OR "myanma"[Text Word] OR "burma"[Text Word] OR "namibia"[Text Word] OR "nepal"[Text Word] OR "netherlands antilles"[Text Word] OR "new caledonia"[Text Word] OR "nicaragua"[Text Word] OR "niger"[Text Word] OR "nigeria"[Text Word] OR "northern mariana islands"[Text Word] OR "oman"[Text Word] OR "muscat"[Text Word] OR "pakistan"[Text Word] OR "palau"[Text Word] OR "palestine"[Text Word] OR "panama"[Text Word] OR "paraguay"[Text Word] OR "peru"[Text Word] OR "philippines"[Text Word] OR "philipines"[Text Word] OR "phillipines"[Text Word] OR "phillippines"[Text Word] OR "poland"[Text Word] OR "portugal"[Text Word] OR "puerto rico"[Text Word] OR "rhodesia"[Text Word] OR "romania"[Text Word] OR "rumania"[Text Word] OR "roumania"[Text Word] OR "russia"[Text Word] OR "russian"[Text Word] OR "rwanda"[Text Word] OR "ruanda"[Text Word] OR "saint kitts"[Text Word] OR "st kitts"[Text Word] OR "nevis"[Text Word] OR "saint lucia"[Text Word] OR "st lucia"[Text Word] OR "saint vincent"[Text Word] OR "st vincent"[Text Word] OR "grenadines"[Text Word] OR "samoa"[Text Word] OR "samoan islands"[Text Word] OR "sao tome"[Text Word] OR "saudi arabia"[Text Word] OR "senegal"[Text Word] OR "serbia"[Text Word] OR "montenegro"[Text Word] OR "seychelles"[Text Word] OR "sierra leone"[Text Word] OR "slovenia"[Text Word] OR "sri lanka"[Text Word] OR "ceylon"[Text Word] OR "solomon islands"[Text Word] OR "somalia"[Text Word] OR "sudan"[Text Word] OR "suriname"[Text Word] OR "surinam"[Text Word] OR "swaziland"[Text Word] OR "syria"[Text Word] OR "tajikistan"[Text Word] OR "tadzhikistan"[Text Word] OR "tadjikistan"[Text Word] OR "tadzhik"[Text Word] OR "tanzania"[Text Word] OR "thailand"[Text Word] OR "togo"[Text Word] OR "togolese republic"[Text Word] OR "tonga"[Text Word] OR "trinidad"[Text Word] OR "tobago"[Text Word] OR "tunisia"[Text Word] OR "turkey"[Text Word]

	OR "turkmenistan"[Text Word] OR "turkmen"[Text Word] OR "uganda"[Text Word] OR "ukraine"[Text Word] OR "uruguay"[Text Word] OR "ussr"[Text Word] OR "soviet union"[Text Word] OR "union of soviet socialist republics"[Text Word] OR "uzbekistan"[Text Word] OR "vanuatu"[Text Word] OR "new hebrides"[Text Word] OR "venezuela"[Text Word] OR "vietnam"[Text Word] OR "viet nam"[Text Word] OR "west bank"[Text Word] OR "yemen"[Text Word] OR "yugoslavia"[Text Word] OR "zambia"[Text Word] OR "zimbabwe"[Text Word])	
#5	Search #1 AND #2 AND #3 AND #4	17,286

33

Database	Global Health
Platform	Ebsco
Years Searched	1973-present
Date Last Searched	January 21, 2021
Limits	None
Number of Results	4,506
Notes	Altered terms to remove respiratory failure, respiratory insufficiency, respiratory depression

#1	TX ("Acute Respiratory Distress Syndrome" OR "Adenoviridae Infections" OR "Bronchiolitis" OR "Bronchitis" OR "Bronchopneumonia" OR "Coronavirus Infections" OR "coronavirus" OR "COVID-19 " OR "covid" OR "crackles" OR "croup" OR "empyema" OR "epiglottitis" OR "flu" OR "h1n1" OR "Influenza" OR "laryngitis" OR "laryngotracheobronchitis" OR "MERS Virus" OR "MERS-CoV" OR "Middle East respiratory syndrome" OR "mycoplasma pneumoniae" OR "nCov" OR "Orthomyxoviridae Infections" OR "parapneumonic effusion" OR "pertussis" OR "Picornaviridae Infections" OR "pleural effusion" OR "pleurisy" OR "pleuropneumonia" OR "pneumonia" OR "Pneumovirus Infections" OR "pulmonary abscess" OR "pyothorax" OR "rales" OR "respiratory infection" OR "Respiratory Syncytial Virus" OR "respiratory tract infection" OR "respiratory viral infection" OR "rhinitis" OR "rhonchi" OR "RSV" OR "sars-cov2" OR "severe acute respiratory infection" OR "severe acute respiratory syndrome coronavirus 2" OR "stridor" OR "tuburculosis" OR "wheezing" OR "whooping cough")
#2	TX ("acute care" OR "adjuvant" OR "Albuterol" OR "albuterol" OR "anti-biotic" OR "anti-infective" OR "anti-viral" OR "antibiotic" OR "antiviral" OR "artificial respiration" OR "atomizer" OR "bag valve mask" OR "bag-valve mask" OR "blow by" OR "continuous monitoring" OR "continuous positive airway pressure" OR "corticosteroids" OR "CPAP" OR "Critical Care" OR "drug" OR "Emergencies" OR "Emergency Medical Services" OR "Emergency Medicine" OR "Emergency Treatment" OR "emergency" OR "Emergicenters" OR "face down" OR "face mask" OR "face-down" OR "inhaler" OR "intubation" OR "mechanical ventilation" OR "medication" OR "Micronutrients" OR "nasal cannula" OR "nebulizer" OR "non-invasive ventilation" OR "noninvasive ventilation" OR "Oximetry" OR "Oxygen Inhalation Therapy" OR "oxygen tent" OR "oxygen therapy" OR "positioning" OR "Posture" OR "pre-hospital" OR "prehospital" OR "prone" OR "pulse

	oximetry" OR "Respiratory support" OR "Respiratory System Agents" OR "Respiratory therapy" OR "risk stratification" OR "risk-stratify" OR "salbutamol" OR "side lying" OR "side-lying" OR "steroid" OR "supine" OR "supplemental oxygen" OR "supportive care" OR "therapeutic" OR "triage" OR "upright" OR "vaporizer" OR "vitamin" OR "zinc")
#3	TX ("Multicenter Study" OR "case comparison" OR "case control" OR "case controlled" OR "case referent" OR "Case-Comparison" OR "controlled clinical trial" OR "Observational Study " OR "Placebo" OR "randomised" OR "randomized" OR "randomized controlled trial" OR "retrospective")
#4	TX (afghanistan OR albania OR algeria OR "american samoa" OR angola OR "antigua and barbuda" OR antigua OR barbuda OR argentina OR armenia OR armenian OR aruba OR azerbaijan OR bahrain OR bangladesh OR barbados OR "republic of belarus" OR belarus OR byelarus OR belorussia OR byelorussian OR belize OR "british honduras" OR benin OR dahomey OR bhutan OR bolivia OR "bosnia and herzegovina" OR bosnia OR herzegovina OR botswana OR bechuanaland OR brazil OR brasil OR bulgaria OR "burkina faso" OR "burkina fasso" OR "upper volta" OR burundi OR urundi OR "cabo verde" OR "cape verde" OR cambodia OR kampuchea OR "khmer republic" OR cameroon OR cameron OR cameroun OR "central african republic" OR "ubangi shari" OR chad OR chile OR china OR colombia OR comoros OR "comoro islands" OR "iles comores" OR mayotte OR "democratic republic of the congo" OR "democratic republic congo" OR congo OR zaire OR "costa rica" OR "cote d’ivoire" OR "cote d’ ivoire" OR "cote divoire" OR "cote d ivoire" OR "ivory coast" OR croatia OR cuba OR cyprus OR "czech republic" OR czechoslovakia OR djibouti OR "french somaliland" OR dominica OR "dominican republic" OR ecuador OR egypt OR "united arab republic" OR "el salvador" OR "equatorial guinea" OR "spanish guinea" OR eritrea OR estonia OR eswatini OR swaziland OR ethiopia OR fiji OR gabon OR "gabonese republic" OR gambia OR "georgia (republic)" OR georgia OR georgian OR ghana OR "gold coast" OR gibraltar OR greece OR grenada OR guam OR guatemala OR guinea OR "guinea bissau" OR guyana OR "british guiana" OR haiti OR hispaniola OR honduras OR hungary OR india OR indonesia OR timor OR iran OR iraq OR "isle of man" OR jamaica OR jordan OR kazakhstan OR kazakh OR kenya OR "democratic people’s republic of korea" OR "republic of korea" OR north korea OR south korea OR korea OR kosovo OR kyrgyzstan OR kirghizia OR kirgizstan OR "kyrgyz republic" OR kirghiz OR laos OR "lao pdr" OR "lao people's democratic republic" OR latvia OR lebanon OR "lebanese republic" OR lesotho OR basutoland OR liberia OR libya OR "libyan arab jamahiriya" OR lithuania OR macau OR macao OR "republic of north macedonia" OR macedonia OR madagascar OR "malagasy republic" OR malawi OR nyasaland OR malaysia OR "malay federation" OR "malaya federation" OR maldives OR "indian ocean islands" OR "indian ocean" OR mali OR malta OR micronesia OR "federated states of micronesia" OR kiribati OR "marshall islands" OR nauru OR "northern mariana islands" OR palau OR tuvalu OR mauritania OR mauritius OR mexico OR moldova OR moldovian OR mongolia OR montenegro OR morocco OR ifni OR mozambique OR "portuguese east africa" OR myanmar OR burma OR namibia OR nepal OR "netherlands antilles" OR nicaragua OR niger OR nigeria OR oman OR muscat OR pakistan OR panama OR "papua new guinea" OR paraguay OR peru OR philippines OR philipines OR phillipines OR phillippines OR poland OR "polish people's republic" OR portugal OR "portuguese republic" OR "puerto rico" OR romania OR russia OR "russian federation" OR ussr OR "soviet union" OR "union of soviet socialist republics" OR rwanda OR ruanda OR samoa OR "pacific islands" OR polynesia OR "samoan islands" OR "navigator island" OR "navigator islands" OR "sao tome and principe" OR "saudi arabia" OR senegal OR serbia OR seychelles OR "sierra leone" OR slovakia OR "slovak republic" OR

slovenia OR melanesia OR "solomon island" OR "solomon islands" OR "norfolk island" OR "norfolk islands" OR somalia OR "south africa" OR "south sudan" OR "sri lanka" OR ceylon OR "saint kitts and nevis" OR "st. kitts and nevis" OR "saint lucia" OR "st. lucia" OR "saint vincent and the grenadines" OR "saint vincent" OR "st. vincent" OR grenadines OR sudan OR suriname OR surinam OR "dutch guiana" OR "netherlands guiana" OR syria OR "syrian arab republic" OR tajikistan OR tadjikistan OR tadzhikistan OR tadzhik OR tanzania OR tanganyika OR thailand OR siam OR "timor leste" OR "east timor" OR togo OR "togolese republic" OR tonga OR "trinidad and tobago" OR trinidad OR tobago OR tunisia OR turkey OR turkmenistan OR turkmen OR uganda OR ukraine OR uruguay OR uzbekistan OR uzbek OR vanuatu OR "new hebrides" OR venezuela OR vietnam OR "viet nam" OR "middle east" OR "west bank" OR gaza OR palestine OR yemen OR yugoslavia OR zambia OR zimbabwe OR "northern rhodesia" OR "global south" OR "africa south of the sahara" OR "sub saharan africa" OR "subsaharan africa" OR "africa, central" OR "central africa" OR "africa, northern" OR "north africa" OR "northern africa" OR magreb OR maghrib OR sahara OR "africa, southern" OR "southern africa" OR "africa, eastern" OR "east africa" OR "eastern africa" OR "africa, western" OR "west africa" OR "western africa" OR "west indies" OR "indian ocean islands" OR caribbean OR "central america" OR "latin america" OR "south and central america" OR "south america" OR "asia, central" OR "central asia" OR "asia, northern" OR "north asia" OR "northern asia" OR "asia, southeastern" OR "southeastern asia" OR "south eastern asia" OR "southeast asia" OR "south east asia" OR "asia, western" OR "western asia" OR "europe, eastern" OR "east europe" OR "eastern europe" OR "developing country" OR "developing countries" OR "developing nation" OR "developing nations" OR "developing population" OR "developing populations" OR "developing world" OR "less developed country" OR "less developed countries" OR "less developed nation" OR "less developed nations" OR "less developed population" OR "less developed populations" OR "less developed world" OR "lesser developed country" OR "lesser developed countries" OR "lesser developed nation" OR "lesser developed nations" OR "lesser developed population" OR "lesser developed populations" OR "lesser developed world" OR "under developed country" OR "under developed countries" OR "under developed nation" OR "under developed nations" OR "under developed population" OR "under developed populations" OR "under developed world" OR "underdeveloped country" OR "underdeveloped countries" OR "underdeveloped nation" OR "underdeveloped nations" OR "underdeveloped population" OR "underdeveloped populations" OR "underdeveloped world" OR "middle income country" OR "middle income countries" OR "middle income nation" OR "middle income nations" OR "middle income population" OR "middle income populations" OR "low income country" OR "low income countries" OR "low income nation" OR "low income nations" OR "low income population" OR "low income populations" OR "lower income country" OR "lower income countries" OR "lower income nation" OR "lower income nations" OR "lower income population" OR "lower income populations" OR "underserved country" OR "underserved countries" OR "underserved nation" OR "underserved nations" OR "underserved population" OR "underserved populations" OR "underserved world" OR "under served country" OR "under served countries" OR "under served nation" OR "under served nations" OR "under served population" OR "under served populations" OR "under served world" OR "deprived country" OR "deprived countries" OR "deprived nation" OR "deprived nations" OR "deprived population" OR "deprived populations" OR "deprived world" OR "poor country" OR "poor countries" OR "poor nation" OR "poor nations" OR "poor population" OR "poor populations" OR "poor world" OR "poorer country" OR "poorer countries" OR "poorer nation" OR "poorer nations" OR "poorer population" OR "poorer populations" OR "poorer world" OR "developing

	economy" OR "developing economies" OR "less developed economy" OR "less developed economies" OR "lesser developed economy" OR "lesser developed economies" OR "under developed economy" OR "under developed economies" OR "underdeveloped economy" OR "underdeveloped economies" OR "middle income economy" OR "middle income economies" OR "low income economy" OR "low income economies" OR "lower income economy" OR "lower income economies" OR "low gdp" OR "low gnp" OR "low gross domestic" OR "low gross national" OR "lower gdp" OR "lower gnp" OR "lower gross domestic" OR "lower gross national" OR lmic OR lmics OR "third world" OR "lami country" OR "lami countries" OR "transitional country" OR "transitional countries" OR "emerging economies" OR "emerging nation" OR "emerging nations")	
#5	Search #1 AND #2 AND #3 AND #4	4,506

Database	WHO Global Index Medicus
Platform	https://www.globalindexmedicus.net/
Years Searched	
Date Last Searched	January 21, 2021
Limits	None
Number of Results	3,388
Notes	Date coverage is not available

#1	(tw:("Acute Respiratory Distress Syndrome" OR "Adenoviridae Infections" OR "Bronchiolitis" OR "Bronchitis" OR "Bronchopneumonia" OR "Coronavirus Infections" OR "coronavirus" OR "COVID-19 " OR "covid" OR "crackles" OR "croup" OR "empyema" OR "epiglottitis" OR "flu" OR "h1n1" OR "Influenza" OR "laryngitis" OR "laryngotracheobronchitis" OR "MERS Virus" OR "MERS-CoV" OR "Middle East respiratory syndrome" OR "mycoplasma pneumoniae" OR "nCov" OR "Orthomyxoviridae Infections" OR "parapneumonic effusion" OR "pertussis" OR "Picornaviridae Infections" OR "pleural effusion" OR "pleurisy" OR "pleuropneumonia" OR "pneumonia" OR "Pneumovirus Infections" OR "pulmonary abscess" OR "pyothorax" OR "rales" OR "respiratory infection" OR "Respiratory Syncytial Virus" OR "respiratory tract infection" OR "respiratory viral infection" OR "rhinitis" OR "rhonchi" OR "RSV" OR "sars-cov2" OR "severe acute respiratory infection" OR "severe acute respiratory syndrome coronavirus 2" OR "stridor" OR "tuburculosis" OR "wheezing" OR "whooping cough")	
#2	(tw:("acute care" OR "adjuvant" OR "Albuterol" OR "albuterol" OR "anti-biotic" OR "anti-infective" OR "anti-viral" OR "antibiotic" OR "antiviral" OR "artificial respiration" OR "atomizer" OR "bag valve mask" OR "bag-valve mask" OR "blow by" OR "continuous monitoring" OR "continuous positive airway pressure" OR "corticosteroids" OR "CPAP" OR "Critical Care" OR "drug" OR "Emergencies" OR "Emergency Medical Services" OR "Emergency Medicine" OR "Emergency Treatment" OR "emergency" OR "Emergicenters" OR "face down" OR "face mask" OR "face-down" OR "inhaler" OR "intubation" OR "mechanical ventilation" OR "medication" OR "Micronutrients" OR "nasal cannula" OR "nebulizer" OR "non-invasive ventilation" OR "noninvasive ventilation" OR "Oximetry" OR "Oxygen Inhalation Therapy" OR "oxygen	

	tent" OR "oxygen therapy" OR "positioning" OR "Posture" OR "pre-hospital" OR "prehospital" OR "prone" OR "pulse oximetry" OR "Respiratory support" OR "Respiratory System Agents" OR "Respiratory therapy" OR "risk stratification" OR "risk-stratify" OR "salbutamol" OR "side lying" OR "side-lying" OR "steroid" OR "supine" OR "supplemental oxygen" OR "supportive care" OR "therapeutic" OR "triage" OR "upright" OR "vaporizer" OR "vitamin" OR "zinc")
#3	(tw:("Multicenter Study" OR "case comparison" OR "case control" OR "case controlled" OR "case referent" OR "Case-Comparison" OR "controlled clinical trial" OR "Observational Study " OR "Placebo" OR "randomised" OR "randomized" OR "randomized controlled trial" OR "retrospective")
#4	(tw:(afghanistan OR albania OR algeria OR "american samoa" OR angola OR "antigua and barbuda" OR antigua OR barbuda OR argentina OR armenia OR armenian OR aruba OR azerbaijan OR bahrain OR bangladesh OR barbados OR "republic of belarus" OR belarus OR byelarus OR belorussia OR byelorussian OR belize OR "british honduras" OR benin OR dahomey OR bhutan OR bolivia OR "bosnia and herzegovina" OR bosnia OR herzegovina OR botswana OR bechuanaland OR brazil OR brasil OR bulgaria OR "burkina faso" OR "burkina fasso" OR "upper volta" OR burundi OR urundi OR "cabo verde" OR "cape verde" OR cambodia OR kampuchea OR "khmer republic" OR cameroon OR cameron OR cameroun OR "central african republic" OR "ubangi shari" OR chad OR chile OR china OR colombia OR comoros OR "comoro islands" OR "iles comores" OR mayotte OR "democratic republic of the congo" OR "democratic republic congo" OR congo OR zaire OR "costa rica" OR "cote d'ivoire" OR "cote d' ivoire" OR "cote divoire" OR "cote d ivoire" OR "ivory coast" OR croatia OR cuba OR cyprus OR "czech republic" OR czechoslovakia OR djibouti OR "french somaliland" OR dominica OR "dominican republic" OR ecuador OR egypt OR "united arab republic" OR "el salvador" OR "equatorial guinea" OR "spanish guinea" OR eritrea OR estonia OR eswatini OR swaziland OR ethiopia OR fiji OR gabon OR "gabonese republic" OR gambia OR "georgia (republic)" OR georgia OR georgian OR ghana OR "gold coast" OR gibraltar OR greece OR grenada OR guam OR guatemala OR guinea OR "guinea bissau" OR guyana OR "british guiana" OR haiti OR hispaniola OR honduras OR hungary OR india OR indonesia OR timor OR iran OR iraq OR "isle of man" OR jamaica OR jordan OR kazakhstan OR kazakh OR kenya OR "democratic people's republic of korea" OR "republic of korea" OR north korea OR south korea OR korea OR kosovo OR kyrgyzstan OR kirghizia OR kirgizstan OR "kyrgyz republic" OR kirghiz OR laos OR "lao pdr" OR "lao people's democratic republic" OR latvia OR lebanon OR "lebanese republic" OR lesotho OR basutoland OR liberia OR libya OR "libyan arab jamahiriya" OR lithuania OR macau OR macao OR "republic of north macedonia" OR macedonia OR madagascar OR "malagasy republic" OR malawi OR nyasaland OR malaysia OR "malay federation" OR "malaya federation" OR maldives OR "indian ocean islands" OR "indian ocean" OR mali OR malta OR micronesia OR "federated states of micronesia" OR kiribati OR "marshall islands" OR nauru OR "northern mariana islands" OR palau OR tuvalu OR mauritania OR mauritius OR mexico OR moldova OR moldovian OR mongolia OR montenegro OR morocco OR ifni OR mozambique OR "portuguese east africa" OR myanmar OR burma OR namibia OR nepal OR "netherlands antilles" OR nicaragua OR niger OR nigeria OR oman OR muscat OR pakistan OR panama OR "papua new guinea" OR paraguay OR peru OR philippines OR philipines OR phillipines OR phillippines OR poland OR "polish people's republic" OR portugal OR "portuguese republic" OR "puerto rico" OR romania OR russia OR "russian federation" OR ussr OR "soviet union" OR "union of soviet socialist republics" OR rwanda OR ruanda OR samoa OR "pacific islands" OR polynesia OR "samoan islands" OR "navigator island" OR "navigator islands" OR "sao tome and

principe" OR "saudi arabia" OR senegal OR serbia OR seychelles OR "sierra leone" OR slovakia OR "slovak republic" OR slovenia OR melanesia OR "solomon island" OR "solomon islands" OR "norfolk island" OR "norfolk islands" OR somalia OR "south africa" OR "south sudan" OR "sri lanka" OR ceylon OR "saint kitts and nevis" OR "st. kitts and nevis" OR "saint lucia" OR "st. lucia" OR "saint vincent and the grenadines" OR "saint vincent" OR "st. vincent" OR grenadines OR sudan OR suriname OR surinam OR "dutch guiana" OR "netherlands guiana" OR syria OR "syrian arab republic" OR tajikistan OR tadjikistan OR tadzhikistan OR tadzhik OR tanzania OR tanganyika OR thailand OR siam OR "timor leste" OR "east timor" OR togo OR "togolese republic" OR tonga OR "trinidad and tobago" OR trinidad OR tobago OR tunisia OR turkey OR turkmenistan OR turkmen OR uganda OR ukraine OR uruguay OR uzbekistan OR uzbek OR vanuatu OR "new hebrides" OR venezuela OR vietnam OR "viet nam" OR "middle east" OR "west bank" OR gaza OR palestine OR yemen OR yugoslavia OR zambia OR zimbabwe OR "northern rhodesia" OR "global south" OR "africa south of the sahara" OR "sub saharan africa" OR "subsaharan africa" OR "africa, central" OR "central africa" OR "africa, northern" OR "north africa" OR "northern africa" OR magreb OR maghrib OR sahara OR "africa, southern" OR "southern africa" OR "africa, eastern" OR "east africa" OR "eastern africa" OR "africa, western" OR "west africa" OR "western africa" OR "west indies" OR "indian ocean islands" OR caribbean OR "central america" OR "latin america" OR "south and central america" OR "south america" OR "asia, central" OR "central asia" OR "asia, northern" OR "north asia" OR "northern asia" OR "asia, southeastern" OR "southeastern asia" OR "south eastern asia" OR "southeast asia" OR "south east asia" OR "asia, western" OR "western asia" OR "europe, eastern" OR "east europe" OR "eastern europe" OR "developing country" OR "developing countries" OR "developing nation" OR "developing nations" OR "developing population" OR "developing populations" OR "developing world" OR "less developed country" OR "less developed countries" OR "less developed nation" OR "less developed nations" OR "less developed population" OR "less developed populations" OR "less developed world" OR "lesser developed country" OR "lesser developed countries" OR "lesser developed nation" OR "lesser developed nations" OR "lesser developed population" OR "lesser developed populations" OR "lesser developed world" OR "under developed country" OR "under developed countries" OR "under developed nation" OR "under developed nations" OR "under developed population" OR "under developed populations" OR "under developed world" OR "underdeveloped country" OR "underdeveloped countries" OR "underdeveloped nation" OR "underdeveloped nations" OR "underdeveloped population" OR "underdeveloped populations" OR "underdeveloped world" OR "middle income country" OR "middle income countries" OR "middle income nation" OR "middle income nations" OR "middle income population" OR "middle income populations" OR "low income country" OR "low income countries" OR "low income nation" OR "low income nations" OR "low income population" OR "low income populations" OR "lower income country" OR "lower income countries" OR "lower income nation" OR "lower income nations" OR "lower income population" OR "lower income populations" OR "underserved country" OR "underserved countries" OR "underserved nation" OR "underserved nations" OR "underserved population" OR "underserved populations" OR "underserved world" OR "under served country" OR "under served countries" OR "under served nation" OR "under served nations" OR "under served population" OR "under served populations" OR "under served world" OR "deprived country" OR "deprived countries" OR "deprived nation" OR "deprived nations" OR "deprived population" OR "deprived populations" OR "deprived world" OR "poor country" OR "poor countries" OR "poor nation" OR "poor nations" OR "poor population" OR "poor populations" OR "poor world" OR "poorer country" OR "poorer countries" OR "poorer

	nation" OR "poorer nations" OR "poorer population" OR "poorer populations" OR "poorer world" OR "developing economy" OR "developing economies" OR "less developed economy" OR "less developed economies" OR "lesser developed economy" OR "lesser developed economies" OR "under developed economy" OR "under developed economies" OR "underdeveloped economy" OR "underdeveloped economies" OR "middle income economy" OR "middle income economies" OR "low income economy" OR "low income economies" OR "lower income economy" OR "lower income economies" OR "low gdp" OR "low gnp" OR "low gross domestic" OR "low gross national" OR "lower gdp" OR "lower gnp" OR "lower gross domestic" OR "lower gross national" OR lmic OR lmics OR "third world" OR "lami country" OR "lami countries" OR "transitional country" OR "transitional countries" OR "emerging economies" OR "emerging nation" OR "emerging nations")	
#5	Search #1 AND #2 AND #3 AND #4	3,388

Summary

Total references retrieved	25,180
# Duplicates removed in EndNote (prior to Covidence upload)	4,462
# Duplicates detected in Covidence:	70
Total # references added to Title/Abstract Screening	20,648

Table S1. Summary of Findings – Respiratory Support and Supportive Care.

Study ID	Country	Design	Age	Study Aim	N	Setting	Intervention	Key Findings	RoB
**Respiratory support (n=14)**									
**Oxygen delivery system (n=8)**									
Pneumonia and ALRTI									
Duke 2001	Papua New Guinea	Prospective cohort	1-59 months	To investigate the severity and duration of hypoxemia in children with severe or very severe pneumonia, to study the predictive value of clinical signs for severity of hypoxemia, the predictive value of SpO2 and other variables for mortality	961	Hospital Ward	Oxygen monitoring protocol (admission and daily) with administration of O2 if SaO2<85%	The mortality rate ratio between this retrospective control group and the 703 children managed with pulse oximetry was 0.65 (95%CI 0.41,1.02, Fisher's exact test, P= 0.07).	Moderate
Duke 2008	Papua New Guinea	Pre-/Post-Study	Not stated	To evaluate the effect of an improved oxygen system on death rate in children with pneumonia in Papua New Guinea	11,291	Hospital Ward	Access to Oxygen concentrators, guideline for O2 according to SpO2 and signs, standardized O2-weaning guideline	Risk of death for a child with pneumonia was 35% lower than was that before the project began (risk ratio 0.65 [0.52,0.78], p<0.0001)	
Graham 2019	Nigeria	RCT	<15 years	To evaluate the effects of an improved oxygen system on mortality and clinical practices for hospitalized children with acute lower respiratory tract infection	48,976	Hospital Ward	Multifaceted oxygen system at hospital level 1) standardized oxygen equipment; 2) clinical education and support; 3) technical training and support, and 4) infrastructure and systems support versus pulse oximetry introduction versus baseline	No association with mortality for oxygen system versus pulse oximetry for children with ALRTI (aOR1.09; 95% CI 0.50-2.41; p=0.824). Increased risk of death for neonates overall (aOR1.45; 95% CI 1.04-2.00; p=0.026) but not preterm/low-birth-weight neonates (aOR1.30; 95%CI 0.76-2.23; p=0.366). In secondary analyses, pulse oximetry associated with lower odds of death from Vaseline for children with ALRI (aOR 0.33; 95%CI 0.12-0.92; p=0.035) but not for children, preterm neonates, or neonates overall.	Some
Mulondo 2020	Uganda	RCT	<59 months	To design and optimize an oxygen-sparing nasal reservoir cannula (OSNRC) and collect pilot data on its safety and efficacy among Ugandan	16	Hospital Ward	Oxygen via OSNRC (oxygen sparing nasal reservoir cannula) for 1 hour versus	There were no significant differences between OSNRC and SNC with respect to clinical adverse events, lactate levels, pH, and SpO2. The OSNRC group had a higher mean SpO2than the SNC group (adjusted mean difference, 1.4, 95% confidence	Some

				children hospitalized with hypoxemic pneumonia.			standard nasal cannula	interval 1.1 to 1.8), showing oxygen delivery enhancement	
Muhe 1997	Ethiopia	RCT	0.5-60 months	To compare the frequency of complications when nasopharyngeal catheters or nasal prongs are used to deliver oxygen to children with ALRI.	121	Hospital Ward	Oxygen delivery by nasopharyngeal catheter vs nasal prongs	No difference in hypoxemic episodes. e.g., Mean number of hypoxemic episodes of 2.97 (catheter) vs 2.39 (prongs) for first 24h. No differences in nasal blockage, mucus production or nursing effort required. Ulceration/bleeding of nose more common in catheter group (19.7 versus 6.7%, p<0.05).	Some
Muhe 1998	Ethiopia	RCT	0.5-60 months	To estimate the frequency of complications when nasal catheters or nasal prongs are used to deliver oxygen to children	99	Hospital Ward	Nasal catheter vs nasal prongs for oxygen delivery	No significant differences in complications, mean flow rate or mean episodes of hypoxemia between the control group and the treatment group (2 vs 1; 0.95 vs 0.9; 3 vs 2; no p values reported). Children in the treatment group had more mucus production on Days 1 and 2 than the control group (37% and 29% vs 13% and 11%, p < 0.05). Amount of nursing time required was significantly higher for the treatment group than the control group (max 22 minutes vs 14 minutes, p<0.05).	High
Acute respiratory distress									
Hoffman 2019	South Africa	Pre-post	<13 years	To describe the outcomes of and adverse events related to HFNC in the first year of its use in a level 2 (L2) general pediatric ward, and to compare these outcomes with those of a historical cohort when HFNC was unavailable.	120	Hospital Ward	HFNC oxygen <10 kg: 2.0 L/kg/min and >10 kg: 2.0 L/kg/min for the first 10 kg plus 0.5 L/kg/min for each kg above 10 kg, with a maximum flow rate of 50 L/min	No difference in mortality rate (3.0% vs. 3.7% pre-HFNC and HFNC availability groups) although underpowered for mortality outcome. In the pre-HFNC group 6.5% were transferred to L3; of those 70.4% needed CPAP or invasive ventilation. In the HFNC-availability period, 5.5% were transferred to L3, of those 89.4% received CPAP or invasive ventilation. No serious adverse effects in HFNC group.	Moderate
Kumar 1997	India	Prospective cohort	0-60 months	To compare the efficacy and acceptability of four commonly used oxygen delivery systems	80	Hospital Ward	Step wise approach to O2 administration via head box, facemask, NC, and twin-based pre-nasal catheter	More children achieved paO2 > 90mmHg with head box (69%), vs face mask (57%), vs nasopharyngeal catheter (26 %), twin-holed pre-nasal catheter (25%)	Low
**CPAP, including bubble (n=6)**									
Pneumonia									
Chisti 2015	Banglade sh	RCT	<59 months	To evaluate whether oxygen therapy delivered by bubble continuous positive airway pressure (CPAP) improved	225	Intensiv e Care Unit	Bubble CPAP versus high-flow (2L/kg per minute up to max of 12 L/min) versus	Lower rates of death (4% vs. 15%; RR 0.25; 95% CI 0.07-0.89; p=0.022) and treatment failure in bCPAP vs low-flow oxygen group ([RR] 0.27, 99.7% CI 0.7,0.99; p=0.0026). No difference in treatment failure was noted	Some

				outcomes compared with standard low-flow and high-flow oxygen therapies			standard low-flow nasal cannula (2L/min)	between patients in the bubble CPAP and those in the high-flow oxygen group (RR 0.50, 99.7% 0.11,2.29; p=0.175).	
McCollum 2019	Malawi	RCT	1-59 months	To evaluate whether bubble CPAP improves severe pneumonia mortality in children with high-risk conditions	664	Hospital Ward	1:1 to low-flow nasal cannula oxygen or nasal bCPAP	Greater relative risk of hospital death (17% vs 11%; RR 1.52, 95% CI 1.02,2.27; p=0.036) with bCPAP vs low-flow oxygen. The RR of 2-week hospital treatment failure in the treatment group was 1.32 (95% CI 0.96,1.83); p=0.081). There was no significant difference in 30-day post discharge mortality in the two groups.	Some
Acute respiratory distress									
Machen 2015	Malawi	Prospective cohort	8-416 days	To describe the outcomes of infants and young children with respiratory distress when treated with a novel, low-cost, stand-alone bubble Continuous Positive Airway Pressure (bCPAP) system in a resource-limited setting	75	Hospital Ward	Bubble CPAP	All physicians surveyed stated that bubble CPAP was useful and led to a change in clinical practice; 80% of survivors had a lower Respiratory Index of Severity in Children (RISC) score score 24 hours after starting bubble CPAP	Moderate
Wilson 2013	Ghana	RCT	3-60 months	To evaluate the effectiveness of immediate versus delayed CPAP in children presenting with acute respiratory distress	70	ED / A&E	Immediate vs delayed CPAP	Mean respiratory rate of children who received immediate CPAP fell by 16 breaths/min (95% CI 10-21) in the first hour compared with no change in children who had CPAP delayed by 1 hour (95% CI2 to +5) p<0.001	Low
Wilson 2017	Ghana	RCT	1-60 months	To determine if continuous positive airway pressure (CPAP), a form of non-invasive ventilation, decreases all-cause mortality in children with undifferentiated respiratory distress in Ghana	2,200	ED / A&E	Nasal CPAP	No difference in 2-week mortality in unadjusted analysis; 26 (3%) of 1021 patients in the CPAP group, and 44 (4%) of 1160 patients in the control group, had died (relative risk [RR] of mortality 0.67, 95% CI 0.42,1.08; p=0.11. However, in adjusted analysis, in children younger than 1-year, all-cause mortality was ten (3%) of 374 patients in the CPAP group, and 24 (7%) of 359 patients in the control group (RR 0·40, 0·19–0·82; p=0·01).	Low
Bronchiolitis									
Lal 2018	India	RCT	1-12 months	To evaluate the efficacy of nasal continuous positive airway pressure (nCPAP) in decreasing respiratory distress in bronchiolitis	72	Hospital Ward	Oxygen via bubble CPAP for 60 minutes	Improvement in respiratory rate [8.0 (5.8) vs 5.1 (4.0), P=0.02], Silverman-Anderson score [0.78 (0.87) vs 0.39 (0.73), P=0.029] and in Modified Pediatric Society of New Zealand Severity Score [2.5 (3.01) vs. 1.08 (1.3), P=0.012]	Some

								were significantly different in the CPAP and standard care groups, respectively	
**Supportive care (n=5)**									
**Physiotherapy (n=4)**									
**Pneumonia**									
Abdelbasset 2015	Egypt	RCT	1-59 months	To evaluate the effect of chest physiotherapy on children with pneumonia	50	Hospital Ward	Chest physical therapy x20 minutes TID during admission	Shorter time to clinical resolution (4 vs 7 days, p=0.012) and greater reduction in respiratory rate (40 to 30 bpm vs 39 to 34 bpm, p= 0.001) and arterial oxygen saturation (93% to 98% vs 93% to 95%, p=0.002) chest PT group.	Some
Lukrafka 2012	Brazil	RCT	1-12 years	To evaluate the efficacy of chest physiotherapy as an adjuvant treatment in children hospitalized with acute community acquired pneumonia	79	Hospital Ward	Chest physiotherapy for 10-12 minutes TID during hospital admission	No differences between groups in severity score, respiratory rate, or duration of hospitalization between the control and intervention groups (6 vs 8 days, p=0.11, respectively).	Some
**Bronchiolitis**									
Bohe 2004	Argentina	RCT	<24 months	To determine if chest physical therapy is useful in the treatment of bronchiolitis and if it decreases the length of hospitalization	32	Hospital Ward	Chest physiotherapy compared to standard nasal aspiration	No significant difference in severity score at admission (5.56 +/- 1.96 vs 5.75 +/- 1.61, p=0.77) or discharge (3.25+/- 1.27 vs 3.12 +/- 1.15, p=0.76), or in LOS (4.0+/-2 vs 3.87 +/- 1.3, p=0.84) between intervention vs control	Some
Gomes 2012	Brazil	RCT	1-24 months	To evaluate the effectiveness of chest physical therapy (CP) in reducing the clinical score in infants with acute viral bronchiolitis (AVB)	30	Hospital Ward and ICU	1) new Chest PT (nCPT) with prolonged slow expiration (PSE) and clearance rhino pharyngeal retrograde (CRR); 2) convention (cCPT) Chest physical therapy with conventional techniques 3) Suction of the upper airways.	Wang respiratory score on admission was reduced in group 1 (7.0 to 4.0; pre/post) and group 2 (7.5 to 5.5) but was unchanged in group 3 (7.5 to 7.0); observed a change 48 hours after hospitalization in group 1 (5.5 to 3.0) and group 2 (4.0 to 2.0) and in 72 hours, there was a change in group 1 (2.0 to 1.0) compared with cCPT	Some
**Other (n=1)**									
Singh 1990	India	Non-randomized clinical trial	1-36 months	To evaluate effect of steam therapy in acute lower respiratory tract infections	36	ED / A&E	Standard of care, study group also received steam from a steam-kettle in a cloth tent.	No significant difference in duration of respiratory distress in children with pneumonia, though there was some improvement for those with bronchiolitis.	Some

Abbreviations: bCPAP: bubble continuous positive airway pressure; CI: confidence interval; IRR: incidence rate ratio; LOS: length-of-stay

Table S2. Summary of Findings – Medications (N=80).

Study ID	Country	Design	Age	Study Aim	N	Setting	Intervention	Key Findings	RoB
**Antibiotics (n=17)**									
Severe Pneumonia									
Addo-Yobo 2004	Colombia, Ghana, India, Mexico, Pakistan, South Africa, Vietnam, Zambia	RCT	3-59 months	To do a multicenter equivalency study comparing oral amoxicillin with injectable penicillin in the treatment of WHO-defined severe pneumonia in children	1,702	Hospital Ward	Oral Amoxycillin 45mg/kg per day in 3 doses for 48h	Treatment failure was 19% in each group (161 patients, penicillin; 167 amoxicillin; risk difference ,0-4%; 95% CI, 4.2-3.3) at 48 h. Infancy (age 3-11months; odds ratio 2.72, 95% CI 1.95 to 3.79), very fast breathing (1.94,1.42 to 2.65), and hypoxia (1.95, 1.34 to 2.82) at baseline predicted treatment failure by multivariate analysis	Some
Agweyu 2015	Kenya	RCT	2-59 months	To compare amoxicillin and penicillin in a population of Kenyan children with pneumonia	527	Hospital Ward	Oral amoxycillin 40-45mg/kg twice daily (vs IV/IM benzylpenicillin 50, IU/kg 4 times daily) for minimum of 48h	Treatment failure was observed in 20 of 260 (7.7%) and 21 of 261 (8.0%) of patients in the amoxicillin and benzyl penicillin arms, respectively (risk difference, 0.3% [95% confidence interval, ,5.0% to 4.3%]). Treatment failure by day 5 post-enrollment was 11.4% and 11.0% and rising to 13.5% and 16.8% by day 14 in the amoxicillin vs benzyl penicillin groups, respectively.	Some
Asghar 2008	Bangladesh, Ecuador, India, Mexico, Pakistan, Yemen, Zambia	RCT	2-59 months	To evaluate injectable ampicillin/gentamicin compared with chloramphenicol to reduce treatment failure in community acquired very severe pediatric pneumonia	958	Hospital Ward	Injection ampicillin/gentamicin 200mg/kg/d x 4 doses q6h; control chloramphenicol 75 mg/kg/k q8h x 3 doses	Treatment failure higher in chloramphenicol vs. amp/gent group (RR 1.43, 95%CI 1.03-1.97) at day 5 and for secondary outcomes at 48 hours (RR 1.6, 95%CI 1.1-2.5), and day 10 (RR 1.37, 95%CI 1.03-1.83) and 21 days (RR1.34, 95%CI 1.02-1.75)	Low
Cetinkaya 2004	Turkey	RCT	2-24 months	To evaluate the effectiveness of two different antibiotic regimens in the empirical treatment of severe childhood pneumonia	97	Hospital Ward	Ceftriaxone 50mg/kg every 12h vs Pen C (25,000U/kg every 4h plus chloramphenicol (15mg/kg every 6h)	There was no significant difference in cure rates between control group and ceftriaxone group (84.7% vs 80.4%, p >0.05). The number of nurse rounds was much more in control group than ceftriaxone group (80 vs 20, p <0.05).	Some

Duke 2002	Papua New Guinea	RCT	1-59 months	To establish whether the combination of benzylpenicillin and gentamicin or chloramphenicol would be better as first-line treatment in children with severe pneumonia in Papua New Guinea.	1,116	Hospital Ward	Benzylpenicillin (50mg/kg, 6 hourly) and Gentamicin (7.5 mg/kg daily) vs Chloramphenicol (25mg/kg 6-hourly), all IM for at least 5 days.	There was no significant difference in mortality (6% vs. 5%; p=0.35), adverse outcomes (26% vs. 22%; p = 0.11) in chloramphenicol versus penicillin/gentamicin groups.	Some
Hazir 2008	Pakistan	RCT	3-59 months	To determine whether home treatment with high-dose oral amoxicillin and inpatient treatment with parenteral ampicillin were equivalent for the treatment of severe pneumonia in children	2,037	Hospital Ward	Oral amoxicillin syrup (80-90 mg/kg per day in two doses) and sent home (ambulatory group), or IV ampicillin (100 mg/kg per day in four doses) for 48 h as an inpatient (hospitalized group)	87 (8.6%) treatment failures in the hospitalized group and 77 (7.5%) in the ambulatory group (risk difference 1.1%; 95% CI ,1.3 to 3.5) by day 6. Five (0.2%) children died within 14 days of enrolment, one in the ambulatory group and four in the hospitalized group	Some
Jeena 2006	South Africa, Zambia	RCT	3-59 months	To determine whether children aged 3-59 months with mild or non-symptomatic human immunodeficiency virus (HIV) infection and WHO-defined severe pneumonia have a higher failure rate than do HIV-uninfected children when treated with the standard WHO treatment of parenteral penicillin or oral amoxicillin.	464	Hospital Ward	Amoxicillin syrup 45 mg/kg/day in 3 doses or parenteral penicillin 200,000 IU/kg/day in 4 dose for 2 days	Twenty (18.9%) HIV-infected children failed by day 2 compared with 37 (10.3%) uninfected children (adjusted odds ratio (OR) 2.07; 95% confidence interval (CI): 1.07,4.00). Thirty-four (32.1%) HIV-infected children failed treatment by day 14 compared with 76 (21.2%) uninfected children (adjusted OR 1.88; 95% CI: 1.11,3.17). Analysis stratified by age showed that the greatest differential in treatment failure at day 2 and day 14 occurred in the children aged 3-5 months	Low
Ribeiro 2011	Brazil	RCT	2-59 months	To compare clinical response to initial empiric treatment with oxacillin plus ceftriaxone and amoxicillin plus clavulanic acid in hospitalized children diagnosed with very severe community-acquired pneumonia (CAP)	104	Hospital Ward	IV oxacillin at 200 mg/kg/day every 6 hours for 10 days and ceftriaxone IV at 100 mg/kg/day every 12 hours for 10 days versus amoxicillin/clavulanic acid IV at 100 mg/kg/day every 8 hours at the beginning of amoxicillin base treatment	Time to improve tachypnea was less among amoxicillin/clavulanic acid versus oxacillin/ceftriaxone (4.8 ± 2.2 versus 5.8 ± 2.4 days respectively; P = 0.028), as was length of hospital stay (11.0 ± 6.2 versus 14.4 ± 4.5 days respectively; P = 0.002).	Low

Shann 1985	Papua New Guinea	RCT	Not specified	To evaluate whether chloramphenicol plus penicillin is any more effective than chloramphenicol alone	748	Hospital Ward	IM Chloramphenicol 25 mg/kg q 6 h until improvement and then the 25 mg/kg of chloramphenicol PO Q6 h versus Benzylpenicillin IM Q6 h 250 000 units 3-9 kg, and 500 000 u to children 10-19 kg.	No difference in mortality rate between groups (17% chloramphenicol/penicillin vs. 13% chloramphenicol alone).	High
Pneumonia									
Brekhna 2003	Pakistan	RCT	3-72 months	To compare amoxicillin, cefuroxime, clarithromycin in CAP in children	124	Hospital Ward	Group 1: Amoxicillin 75mg/kg/day IV TID vs Group 2: Cefuroxime 75/mg/kg/day IV TID, Group 3: Clarithromycin 15mg/kg/day IV BID	No difference in clinical response between treatment groups as measured by RR (p-0.7), temperature (p = 0.5), oxygen requirement (p = 0.10), inability to feed (p = 0.2)	High
Hasali 2005	Malaysia	RCT	2-59 months	To evaluate the effect of antibiotic regimens used in pediatric community-acquired pneumonia (CAP)management and to conduct a cost-effectiveness analysis (CE) between IV ampicillin versus combination therapy of IV ampicillin and IV gentamicin	40	Hospital Ward	IV ampicillin 100 mg/kg/day divided every 6 h and 5 mg/kg of IV gentamicin as a single daily dose compared to IV ampicillin alone	Shorter antibiotic duration (3.80 ± 1.01 vs 4.80 ± 0.41, P<0.05); Days of hospital stay 3.85 ± 1.10 vs 4.80 ± 0.41 (control vs intervention, p <0.05); time to switch to PO therapy (3.80 ± 1.01 vs 4.80 ± 0.41, p <0.05) in those receiving ampicillin alone.	Some
Mulholland 1995	The Gambia	RCT	0-59 months	To evaluate the effect of chloramphenicol vs. trimethoprim-sulfamethoxazole for the treatment of malnourished children with community-acquired pneumonia	111	Hospital Ward	PO chloramphenicol palmitate 25 mg/kg every 8 hours vs tmp/smx PO 20/200 mg <12 month and 60/300 BID >12-month x 7 days	No difference in number of treatment failures between the treatment group and the control group (16 vs 16)	Some
Straus 1998	Pakistan	RCT	2-59 months	To study effectiveness of co-trimoxazole compared with that amoxicillin in pneumonia therapy, and assessed the clinical impact of co-trimoxazole resistance	595	Hospital Ward	Co-trimoxazole BID (20 mg/kg sulfamethoxazole, 4 mg/kg trimethoprim) versus amoxicillin TID (15 mg/kg per dose)	There were 92 (23%) therapy failures in the co-trimoxazole group and 30 (15%) in the amoxycillin group (p = 0.03) , 26 (13%) versus 12 (12%) among children with non-severe pneumonia (p=0.856) and 66 (33%) versus 18 (18%) among those with severe pneumonia (p= 0.009)	Low

Bronchiolitis									
Kabir 2009	Bangladesh	RCT	<24 months	To ascertain the effect of antibiotics in the management of bronchiolitis	295	Hospital Ward	(i) parenteral ampicillin at 50 mg/kg every 6 hours IV n=99 (ii) oral erythromycin at 10 mg/kg every 6 hours considering Mycoplasma pneumoniae n=99 (iii) no antibiotics n=97	No difference in time to clinical sign recovery (cough, difficulty breathing, wheeze, chest indrawing, tachypnea, tachycardia, rhonchi, crepitation) among three intervention groups (p>0.23, p<0.62, p = 0.54, p <0.27, p = 0.75, p = 0.76, p = 0.81, p >0.98, respectively). Children in no antibiotic group had shorter LOS (3.7 ± 1.5) than parenteral antibiotic group (4.3 ± 1.9) or oral antibiotic group (4.4 ± 1.9) (p<0.001)	Some
Pinto 2012	Brazil	RCT	<12 months	To evaluate if azithromycin reduces the length of hospitalization and oxygen requirement in infants with acute viral bronchiolitis (AB).	184	ED / A&E	Azithromycin 10 mg/kg/d or placebo, administered orally, for 7 days.	The use of azithromycin did not reduce the median number of days of either hospitalization (p= 0.28) or oxygen requirement (p=0.47)	Low
SARI									
Gamiño-Arroyo 2019	Mexico	RCT	>12 months	To evaluate the effectiveness of nitazoxanide (NTZ) for acute respiratory viral illness	257	Hospital Ward	NTZ 600mg for> 12yo, 200mg for 4-11yo, 100mg for 1-3yo; twice daily for 5 days	No difference in hospital LOS (6.5 days in the NTZ group vs 7.0 in the placebo group; p = 0.56). No difference in hospital LOS similar in children (p = 0.29) and adults (p = 0.62).	Low
**Nebulized treatment (n=17)**									
Bronchiolitis									
Adhikari 2016	Nepal	Prospe ctive cohort	2-24 months	To compare initial response of nebulized adrenaline and salbutamol for infants and young children admitted with bronchiolitis	80	Hospital Ward	Adrenaline 0.1 ml/kg 1:1000 nebulization q6h versus Salbutamol nebulization 0.15 mg/kg q6h	No difference in mean RDAI scores over 48 hours to 4.15 (CI 3.57, 4.73) and 4.13 (CI- 3.69, 4.56), hospital LOS (5.32 days vs. 5.68 days) in adrenaline and salbutamol group respectively. Patients nebulized with adrenaline required oxygen for 33.30 hours compared with 36.45 hours in salbutamol.	Serious
Ejaz 2015	Pakistan	RCT	1-24 months	To evaluate the effect of nebulized 3% hypertonic saline or NS on patients with acute bronchiolitis	80	ED / A&E	Nebulized 3% hypertonic saline (3 cc) vs Nebulized NS (3cc)	The HS group had a significantly greater reduction in respiratory score than NS (3.23+/-1.17 vs 2.05 +/- 0.50, p <0.001)	High
Farrah 2014	Pakistan	RCT	2-24 months	To evaluate the effect of nebulized N-acetylcysteine in treatment of bronchiolitis	100	Hospital Ward	Group 1 20mg NAC in 3ml saline nebulization; group-2 received inhalation of 2.5 mg salbutamol in 3 ml saline nebulization. TID x 5 days.	Clinical severity score improved on day 3 (2.90 +/- 1.48 vs 0.88 +/- 1.08) and day 5 (3.30 +/- 1.77 and 1.90 +/- 1.32) in NAC group vs control. Shorter LOS in NAC group 4.36 +/- 1.66 days vs control 4.98 +/- 2.6 days (p = 0.108)	High
Tinsa 2009	Tunisia	RCT	<12 months	To evaluate the efficacy of nebulized terbutaline in bronchiolitis	35	Hospital Ward	Nebulized Terbutaline 0.15mg/kg, repeated at 30 minutes, then every	No significant differences at baseline, 30 minutes, 60 minutes 120 minutes in RDAI	Some

							4 hours for the study period versus nebulized saline	score, O2 sat, RR, HR, or duration of hospitalization for terbutaline versus saline.	
Gadomski 1994	Egypt	RCT	<18 months	To determine the efficacy of albuterol in reducing respiratory distress in infants with bronchiolitis and assess which route of delivery is more effective (nebulization vs oral)	169	Other: Outpatient and casualty department	Albuterol neb 0.15mg/kg per dose 30 minutes apart x 2 doses; oral albuterol 0.15mg/kg per dose x 1 dose; oral placebo; nebulized saline	No difference among four groups in heart rate, respiratory rate, or SpO2 (except for increase in HR in nebulized albuterol group at 60 minutes p = 0.058). However, in recurrent wheezing group nebulized albuterol improved clinical score (8-point decrease, p =0.0003)	Some
Jawaria 2015	Pakistan	RCT	<24 months	To compare the efficacy of normal saline and inhaled beta-agonist in the treatment of bronchiolitis	72	Hospital Ward	Salbutamol nebulization 0.2mg/kg in 2cc NS q4h (group A) vs NS nebulization q4h (group B)	58.33% in Group-A (intervention) and 25% in Group-B were "treated effectively" as measured by reduction in clinical bronchiolitis severity score (p=0.008).	High
Khashabi 2005	Iran	RCT	2-24 months	To compare the efficacy of nebulized epinephrine with salbutamol on infants with severe bronchiolitis	72	ED / A&E	Epinephrine 0.1ml/kg; salbutamol 0.15 mg/kg; normal saline (placebo). Drug mixed with normal saline for total volume 5mL and nebulized x 3 doses each 20 minutes apart	Pre- and post-treatment mean oxygen saturation, clinical score, and respiratory rate significantly different (p <0.00) between the three groups. Mean SaO2 increased from 86.4 to 91.9 in epinephrine and 84.3 to 90.5 in salbutamol groups (p = 0.00 pre- vs. post-intervention), however no significant difference in placebo group for SaO2 86.3 to 88.8 (p=0.1). Pre- and post-intervention clinical score different in all study groups (p = 0.035).	Low
Kumar A 2019	India	RCT	2-24 months	To compare 3% versus 0.9% saline nebulization in children with bronchiolitis	150	Hospital Ward	3 mL Nebulized Saline 3%	At 24 hours, lower mean clinical severity score for control group was 2.49±1.03 and treatment group was 2.16±0.49 (P=0.013). Improvement in O2 saturation in the treatment group was better in the first 24 hours (55% vs 28%, p <0.05). The duration of hospital stay was shorter in treatment group (2.35 days vs 4.04 days, p <0.001).	Some
Modaressi 2012	Iran	RCT	1-24 months	To determine the efficacy of epinephrine compared to salbutamol in treatment of bronchiolitis	40	Hospital Ward	Epinephrine 0.1 mgl/kg vs salbutamol 0.15 mg/kg. Three doses given at 20-minute intervals	Mean hospital duration was 3.3 +/- 1.1 vs 3+/- 0.9 salbutamol vs epinephrine (p=0.03). Significant difference in RDAI between the groups (p = 0.02). No difference in SpO2, heart rate, respiratory rate	Some
Ojha 2014	Nepal	RCT	1.5-24 months	To evaluate the clinical profile and the effect of use of hypertonic (3%) saline vs NS	72	ED / A&E	Nebulized 3% Saline vs NS every 8 hours each day until discharge	No difference between intervention and control. Mean (±SD) for duration of hospital stay was 44.82 (¬±23.15) and 43.60 (±28.25) for 3% and 0.9% group respectively (p = 0.86). Likewise, mean (SD) duration of oxygen supplementation was 32.50 (±20.44) and 34.50 (±26.03) for 3% and 0.9% group respectively (p = 0.85). Moreover, time required for normalization of clinical score was 36.79 (±19.53) and	Low

								38.34 (±26.67) for 3% and 0.9% group respectively (p = 0.80).	
Pukai 2020	Papua New Guinea	RCT	<24 months	To determine if nebulized normal saline vs standard care was beneficial in bronchiolitis	199	ED / A&E	1-3 doses of nebulized saline 2 ml per dose over the first 3 hours in addition to standard care or standard care	Greater reduction in mean respiratory distress score (RDS) at 4 hours by 3.41 (95% CI 3.0-3.8) versus 1.96 (95% CI 1.5-2.4, p< 0.0001), increase in SpO2 at 4 hours (7% vs 4%; p<0.001), and ability to be discharged from ED (58% vs 28.2%; p<0.001) in NS vs standard of care group.	Low
Ray 2002	India	RCT	2-24 months	To assess the efficacy of bronchodilators in wheeze associated respiratory tract infection (WRTI); and (ii) To compare L-adrenaline with salbutamol in WRTI	91	Hospital Ward	L-adrenaline (0.1ml/kg/dose in 1in 10,000 solution) salbutamol (0.1mg/kg/dose) nebulized each at 20 minutes intervals 3 total doses.	Lower mean respiratory rate, RDAI score, clinical score in adrenaline versus salbutamol group, after three doses of nebulization. More patients with improved respiratory distress in adrenaline vs salbutamol (28/44 in Group A vs 12/46in Group B, p <0.001). Fewer patients in adrenaline vs salbutamol (6/45 vs 14/46 requiring admission, p = 0.02)	Some
Shankar 2019	India	Retros pective	6-24 months	To determine if there is significantly shorter duration of stay between children who were given hypertonic saline neb and adrenaline neb.	90	Hospital Ward	Adrenaline nebulization vs hypertonic saline nebulization	Mean duration of stay for children treated with adrenaline nebulization was 5.3 days and those given hypertonic saline was 4.8 days (p = 0.29). No significant difference.	Moderate
Sharma 2013	India	RCT	1-24 months	To compare the length of hospital, stay and improvement in clinical severity scores among children with bronchiolitis nebulized with 3 % hypertonic saline or 0.9% saline	248	Hospital Ward	4 mL 3% hypertonic saline nebulized q4h until discharge	Clinical severity scores monitored afterwards q12h till discharge did not show statistically significant differences in 3% and 0.9% saline groups. Mean length of hospital stays (time to reach predefined clinical severity score<3) was 63.93 ¬± 22.43 h in 3% saline group and 63.51 ¬± 21.27 h in 0.9% saline group (p = 0.878).	Low
Soleimani 2020	Iran	RCT	1-24 months	To investigate the effects of saline usage with different concentrations and salbutamol on the treatment	180	Hospital Ward	Group 1: normal saline 0.9% and 0.15 mg/kg salbutamol nebulizer<br><br>Group 2: 3% and 0.15 mg/kg salbutamol nebulizer<br><br>Group 3: Hypertonic saline 5% and 0.15 mg/kg salbutamol nebulizer	Shorter hospital LOS in Group 2 (4.63 + 1.34, 3.41 + 1.07, and 4.61 + 0.72 days in Group 1, Group 2, Group 3; p<0.001). Decrease in clinical bronchiolitis severity score in Group 1 and Group 3 at 20, 40 and 60 minutes after treatment in both groups (P <0.001) and in Group 2 at 20 and 40 minutes after treatment.	Some
Tinsa 2014	Tunisia	RCT	<12 months	to evaluate the efficacy of nebulized NS vs 5% hypertonic saline alone	94	ED / A&E	NS vs 5% HS vs 5% NS with 0.1% epi nebulized at admission	No significant difference between the 3 groups in severity score, oxygen saturation, clinical signs, or length of stay	Some

				or mixed with epi in bronchiolitis			and every 4 hours throughout hospitalization		
Croup									
Eghbali 2016	Iran	RCT	6 months -6 years	To compare the effect of L-epinephrine plus dexamethasone vs. dexamethasone for treatment of croup in children.	174	Hospital Ward	Single dose L-epinephrine (0.5 mg/kg) with 3ml saline nebulized + Dexamethasone 0.6mg/kg IM vs Dexamethasone alone	Lower mean of Westley clinical croup scores in L-epinephrine vs control group (p <0.009) at 30, 60, 90 min but not 120 minutes	Some
**Nutrients and Minerals (n=17)**									
Vitamin A									
ALRTI / Pneumonia									
Donnen 1998	DRC	RCT	0-72 months	To investigate the effect of 1) single high dose vitamin A; 2) daily, low doses of vitamin A; or 3) placebo on ALRTI and diarrhea in hospitalized preschool-age children	900	Hospital Ward	200 000 IU vitamin A (100 000 IU if aged < 12 mo) PO on the day of admission vs 5000 IU vitamin A until discharge.	Mortality rates were not significantly different among the 3 groups. Low-dose vitamin A showed no significant effect on the duration of moderate or severe diarrhea or on the duration and incidence of ALRIs and all-cause fevers.	Some
Julien 1999	Mozambique	RCT	6-72 months	To evaluate the effect of single high-dose vitamin A supplementation immediately upon admission on recovery in children hospitalized with ALRTI	164	Hospital Ward	200,000 IU vitamin A immediately upon admission (100,000 IU if <1 year)	Median hospital stay 3 in vit A group vs 4 in placebo. Day 5 discharge rate was 99.4% in vit A group vs 73.9% in placebo (p=0.023). No differences between groups at 6-week follow up	Some
Kjolhede 1995	Guatemala	RCT	3-48 months	To test the efficacy of a single high dose of vitamin A as adjuvant treatment for radiographically confirmed cases of ALRTI	263	ED / A&E	200,000 IU Vitamin A (100,000 IU if < 1 year) PO	No difference between groups in the rate of normalization in respiratory rate, oxygen saturation, temperature, or clinical score (p-values not presented). Duration of hospitalization was not different by treatment group. No difference in adverse outcomes (mechanical ventilation, prolonged hospitalization, readmission and death).	Low
Fawzi 1998	Tanzania	RCT	6-60 months	To examine whether large doses of vitamin A given to Tanzanian children hospitalized with non-measles pneumonia would reduce the severity of respiratory disease	687	Hospital Ward	200,000 IU Vitamin A daily x 2 days	No difference in mortality (relative mortality 1.63 95% CI 0.67-0.97; p=0.28) vitamin A vs. placebo. No difference in length of stay (both groups 4.2 days) or time with symptoms.	Low

Hussey 1990	South Africa	RCT	<13 years	To evaluate the effect of Vitamin A on morbidity and mortality in children with severe measles	189	Hospital Ward	40,000 IU Vitamin A PO (1/2 dose on day of admission and 1/2 dose the next day)	Faster recovery in vitamin A vs control group from pneumonia (mean 6.3 vs 12.4 days; p<0.001) and diarrhea (mean 5.6 vs 8.5 days; p<0.001); less croup (13 vs 27 cases; p=0.03); shorter length of hospitalization (mean 10.6 vs 14.8 days; p=0.01). Of 12 children who died 10 were in placebo group (p=0.05) with risk of death lower in Vit A vs control group (RR 0.51, 95%CI 0.35-0.74)	Low
Nacul 1997	Brazil	RCT	6-59 months	To evaluate the impact on clinical recovery and severity of the addition of large doses of vitamin A to the standard treatment for childhood pneumonia.	478	Other: Hospital or outpatie nt	Capsules 100 000 IU of vitamin A and 20 IU of vitamin E in oil. Infants: 1 capsule on admission and next day. 1-4 years: 2 capsules admission and next day	Similar overall duration of pneumonia (median 7.6 days Vit A vs 7.5 days placebo) and incidence of adverse outcomes. Children who received vitamin A, however, were less likely to have fever by day 3 (16% vs 26.4%, P = 0.008) and were 29% less likely to fail to respond to the first line antibiotic (P = 0.054).	Some
Rodriguez 2005	Ecuador	RCT	2-59 months	To evaluate the effect of a moderate dose of vitamin A as an adjunct to standard antimicrobial treatment on the duration of respiratory signs in children with pneumonia	287	Hospital Ward	PO vit A 50,000 to children aged 2-12, and 100000 IU >12-59 months	No difference in the two groups in duration of pneumonia symptoms (tachypnea, fever, hypoxemia).	Some
Si 1997	Vietnam	RCT	1-59 months	To evaluate the effect of high dose vitamin A on morbidity, and if possible, mortality and moderate in children with severe pneumonia in hospitalized Vietnamese children	592	Hospital Ward	Vitamin A 200 000 IU/mL vs placebo A. For children < 1 y the dosage was 0.5ml day of inclusion and the day after. Children aged 1-4y received two doses of 1.0ml. The children allocated to the vitamin A group received in total 200 000 IU and 400 000 IU of retinol, respectively	No differences were found regarding mean time for normalization of fever, respiratory rate and time of hospitalization. However, with stratification for age, sex, nutrition status, moderately malnourished vitamin A-supplemented children to have a shorter time of hospitalization (p = 0.04), due to an effect in females aged > 12 months (p = 0.02) and females with very severe pneumonia (p = 0.048).	Some
Stephensen 1998	Peru	RCT	3 months -10 years	To test the hypothesis that high-dose vitamin A supplements will enhance recovery of children hospitalized for the treatment of community-acquired pneumonia.	95	Hospital Ward	Children <1 year of age received 100 000 IU vitamin A on admission to the hospital and an additional 50 000 IU the next day. Children >1 year of age received 200 000 IU on admission and 100 000 IU the next day	No difference in hospital LOS among two groups. Children receiving vitamin A had lower blood oxygen saturation (the mean difference on day 3 in hospital was 1.1%), higher prevalence rates of retractions (37% in the vitamin A group vs 15% in the placebo group on day 3), auscultatory evidence of consolidation (28% in the vitamin A group vs 17% in the placebo group on day 3) and were more likely to require supplemental oxygen (21% in the vitamin A group vs 8% in the placebo	Low

								group on day 3) than children in the placebo group.	
Vitamin C and E									
Mahalanabis 2006	India	RCT	Not specified	To evaluate Vitamin E and C as adjunct therapy of pneumonia in children with severe ALRTI	85	Hospital Ward	Vitamin E and vitamin C (each 200 mg twice daily for 6 days)	No benefit for intervention group on four clinical outcomes measured (Recovery rate ratios (95% CI) 0.89 (0.64–1.25), 1.01 (0.72–1.41), 0.86 (0.57– 1.29), and 1.12 (0.77–1.64) for very ill status, feeding difficulty, fever, and tachypnea, respectively).	Some
Vitamin D									
Somnath 2017	India	RCT	2-60 months	To study the effect of single oral dose vitamin D3 in decreasing the duration of hospital stay in children hospitalized with ALRI.	154	ED / A&E	Single dose of 100,000 IU of vitamin D3 PO on day one of admission	No difference in median hospital LOS, mortality, PICU admission, complications.	Some
Choudhary 2012	India	RCT	2-59 months	To determine the role of oral vitamin D supplementation for resolution of severe pneumonia in under-five children	200	ED / A&E	Vitamin D 1000 IU <1 year old; 2000 IU children 1-5 years of age, PO within four hours of admission, then once daily x 4 days versus placebo.	No difference in duration of pneumonia, hospital LOS between treatment groups.	Low
Gupta 2016	India	RCT	2-59 months	To evaluate the efficacy of single oral high dose of Vitamin D3 for treatment of pneumonia in under-five children	324	Hospital Ward	Single dose of 100,000 IU Vitamin D x1 PO	No difference in complete recovery from pneumonia, hospital LOS, and fever clearance time between treatment groups.	Low
Rajshekhar 2016	India	RCT	2-59 months	To evaluate the efficacy of vitamin D supplementation in addition to routine treatment of severe pneumonia in children less than 5 years of age	95	Other: both ward and PICU	Vitamin D 1000 IU for children < 1 year and 2000 IU for >1 year versus placebo, within 4 hours after admission, then once daily x 4 days	No significant difference in time to resolution of pneumonia between the two groups (20.8% vs 62.5% resolved in <24 hours, 62.5% vs. 58.5% in 24-48 hours, and 62.5% vs 31.3% in >48 hours; p=0.14).	High
Dhungel 2015	Pakistan	RCT	2-60 months	To see the efficacy of vitamin D in children with vitamin D deficiency with pneumonia	191	Hospital Ward	IM vitamin D 100,000 units within the first 24 hours of admission vs no treatment	No difference in hospital LOS between groups (5.7 days vitamin D vs. 6.1 days placebo, p=0.28).	High
Manaseki-Holland 2010	Afghanistan	RCT	1 week-3 years	To determine whether (i) supplementation of oral 100 000 IU of vitamin D3 on reducing duration of illness in children with pneumonia	453	Other: in hospital or outpatient	100,000 IU cholecalciferol in 1 mL olive oil x1 dose PO	No difference in the mean number of days to recovery between the vitamin D3 (4.74 days; SD 2.22) and placebo arms (4.98 days; SD 2.89; P = 0.17).	Low

**Zinc**									
Bronchiolitis									
Ahadi 2020	Iran	RCT	2-23 months	To compare the effect of zinc gluconate and placebo in the treatment of tachypnea, dyspnea and fever in children aged 2 to 23 months with acute bronchiolitis	100	ED / A&E	1% zinc gluconate PO (1 cc/kg in infants under 1 year and 10cc in infants over one year)	Shorter hospital LOS (4.14±1.21 versus 4.64±1.2 days; p=0.016) in zinc vs placebo group. Less wheezing and rhinorrhea in treatment group at 72 hours (16% vs 36%, p=0.023, 0 vs 12%, p=0.027). No difference between the groups in resolution of fever, tachypnea, nasal flaring, retractions, or cyanosis, at 24 hours or 72 hours.	High
Farhad 2011	Iran	RCT	2-23 months	To evaluate the effect of zinc sulfate in improving the clinical manifestations of acute bronchiolitis in children younger than 2 years	50	Hospital Ward	1ml/kg 1% zinc sulfate (<1 year) PO or 20mg elemental zinc if >1yo	At 24 hours more patients had resolution of tachypnea, retractions, wheezing, cyanosis in control group compared to treatment (p=0.04) otherwise no difference in clinical signs between groups	High
Pneumonia									
Bose 2006	India	RCT	2-23 months	To evaluate the effect of adjuvant zinc therapy on recovery from severe pneumonia by hospitalized children receiving standard antibiotic therapy	299	Hospital Ward	20mg zinc sulfate tablet at enrollment and duration of hospitalization	No clinical or statistically significant differences in the duration of tachypnea, hypoxia, chest indrawing, inability to feed, lethargy, severe illness, or hospitalization. No significant difference in time to recovery (RR 0.93, 95%CI 0.72-1.21, p=0.589), hospital LOS (RR 0.93, 95%CI 0.74, 1.17, p=0.550). No significant differences in symptom resolution.	Low
Brooks 2004	Bangladesh	RCT	2-23 months	To evaluate the effect of zinc on duration of severe pneumonia in children	270	Hospital Ward	20mg Zinc PO per day until hospital discharge, first dose within 1 hour of antibiotics	Shorter duration to resolution 72 vs 96 hours, zinc vs control (HR 0.7, 95%CI 0.51-0.98) and hospital LOS (112 hours both, HR 0.75, 95%CI 0.57-0.99). No differences in resolution of indrawing, hypoxia.	Low
Coles 2007	India	RCT	2-23 months	To explore the effect of etiology on the treatment effect of zinc in young children hospitalized for severe pneumonia	295	Hospital Ward	Zinc: 10mg orally twice a day during hospitalization	Greater median hospital LOS found in Zinc group (87.3h vs 68.5h, p=0.025)	High
Howie 2018	The Gambia	RCT	2-59 months	To assess the benefit of adjunct zinc therapy for children with severe pneumonia	604	Hospital Ward	Oral fixed doses of 10 mg elemental zinc daily for infants and 20 mg daily for children x 7 days	No difference in time to resolution of respiratory symptoms (42.3 vs 30.9 hours respectively; P=0.242) or treatment failure between placebo and zinc arms both on day 5 (14.0% vs 14.1%) and day 10 (5.2% vs 5.9%). Time to recovery from lower chest wall indrawing and sternal retraction was longer in the placebo compared to zinc arm (24.4 vs 23.0 hours; P=0.011 and 18.7 vs 11.0 hours; P=0.006 respectively).	Low

Fataki 2014	Tanzania	RCT	6-36 months	To evaluate the effect of Zinc supplementation for pediatric pneumonia	95	Hospital Ward	12.5mg Zinc sulfate monohydrate in water twice daily until hospital discharge	No difference in hospital LOS (IRR: 0.69; 95% CI 0.45-1.06; p=0.09) or proportion of children who were hospitalized for <3 days (RR: 0.85; 95% CI: 0.57-1.25; p=0.40) or<5 days (RR: 1.01; 95% CI: 0.83-1.23; p=0.92).	Low
Heydarian 2020	Iran	RCT	2-59 months	To evaluate the effect of prescribing zinc sulfate on improving the clinical symptoms of pneumonia in 2-59-month-old children	108	Hospital Ward	oral zinc sulfate 10 mg (1 ml/ kg in children younger than one year, and 20 mg/kg for children above one year every 12 hours.	No difference in hospital LOS between groups (5.7 vs. 5.11 days; p=0.174). Shorter duration of fever between the intervention and control groups 24 and 36 hours after hospitalization (p=0.014, p=0.02) but not at 12 and 48 hours after. Improved tachypnea in the intervention group at 36 hours post-hospitalization (p=0.02) but not during hospitalization or 12, 24, or 48 hours after.	Some
Laghari 2019	Pakistan	RCT	28 days-5 months	To evaluate the efficacy of zinc supplementation in alleviating symptoms and shortening of hospital stay in children with pneumonia	100	Hospital Ward	Oral 20 mg/day zinc supplements vs placebo	No difference in clinical signs between the two groups. Slightly shorter hospital stays in the zinc supplemented group (p=.01)	Some
Mahalanabis 2002	India	RCT	9 months -15 years	To evaluate the effect of zinc supplementation on episodes of illness in children with measles accompanied by pneumonia.	85	Hospital Ward	20mg zinc acetate BID x6 days	No difference in duration until resolution of fever [HR 1.08 (0.67, 1.74)] tachypnea [HR1.26 (0.78, 2.05)], return of appetite [HR0.82 (0.47, 1.44)], or improved or cured status [HR 1.07 (0.64, 1.78)].	Some
Qasemzadeh 2014	Iran	RCT	3-60 months	To investigate the effects of short courses of zinc administration on recovery from this disease in hospitalized children.	120	ED / A&E	Zinc every 12 hours until discharge	Decrease in duration of clinical symptoms (50% vs. 41.7% <2 days symptoms; P=0.044) and hospital LOS (20% vs. 40% LOS > 5 days; P=0.004) in zinc versus control group.	Some
Rerksuppaphol 2020	Thailand	RCT	2-60 months	To assess the efficacy of zinc supplementation on the treatment outcomes of pneumonia.	91	Hospital Ward	15 mg elemental zinc twice daily dissolved in 30 ml distilled water, until their discharge from the hospital, or the completion of7 days.	Shorter hospital LOS (96 vs 144 hours; p=0.008), fever resolution (24 vs 42 hours; p=0.002) and normalization of oxygenation (28 vs 48 hours; p=0.014) in zinc group.	Low
Sempértegui 2014	Ecuador	RCT	2-59 months	To evaluate the effect of zinc given with standard anti-microbial treatment on the duration of respiratory signs in children with severe pneumonia	450	ED / A&E	10 mg elemental zinc (zinc sulfate) in 5 mL syrup BID	No difference in time to resolution of respiratory signs or prevalence of treatment failure (placebo compared with zinc: 34.4% compared with 34.5%; OR: 1.00; 95% CI: 0.68, 1.5)	Low
Shah 2012	Nepal	RCT	2-60 months	To evaluate the efficacy of zinc supplementation	122	Hospital Ward	20 mg zinc sulfate PO or placebo by mouth at	No difference in hospital LOS (p=0.193), severe pneumonia (p=0.219), pneumonia	Some

				in treatment of severe pneumonia in hospitalized children.			enrollment; from day 2, 10 mg zinc twice a day for 7 days.	(p=0.943), no PO intake (p=0.771), IV fluid use (p=0.258), oxygen use (p=0.684) between groups.	
Srinivasan 2012	Uganda	RCT	6-59 months	To determine effect of zinc as adjunct therapy on time to normalization of respiratory rate, temperature and oxygen saturation, and case fatality of severe childhood pneumonia	352	Hospital Ward	zinc (20 mg for children,>12 months, and 10 mg for those < 12months) or a placebo once daily for seven days, in addition to standard antibiotics	Decreased case fatality among group treated with zinc (4.0% in the zinc and 11.9%) in the placebo group with Relative Risk 0.33 (95% CI of 0.15 to 0.76). Excess risk was greater among HIV+ than HIV- children (ARR: 26 (95% CI: 9, 42) per 100 versus 2 (95% CI: -4, 7) per 100). No effect on time to normalization of the respiratory rate, temperature, and oxygen saturation.	Some
Wadhwa 2013	India	RCT	2-24 months	We evaluated the role of zinc as an adjunct to antibiotics in the treatment of children hospitalized for severe or very severe pneumonia	550	Hospital Ward	10 mg zinc PO on admission and every 12 h until recovery or the completion of 14 days.	No difference in time to recovery from severe or very severe pneumonia (HR: 0.98; 95% CI: 0.82, 1.17). In the stratified analysis, zinc was shown to be efficacious in reducing the time to recovery in children with very severe pneumonia (HR: 1.52;95% CI: 1.03, 2.23).	Low
Valentiner-Branth 2010	Nepal	RCT	2-35 months	To measure the effect of zinc supple mentation in children with pneumonia in a population in which zinc deficiency is common.	2,628	Other: Study clinic	10mg zinc sulfate PO or placebo;10 mg for children aged 2-11 mo. and 20 mg for children aged >12 mo then daily x 14 days.	No difference in time to recovery between zinc and placebo groups for non-severe (median: 2 d; hazard ratio: 1.0; 95% CI 0.96-1.1) or severe (median: 4 d; hazard ratio: 1.1; 95% CI 0.79-1.5) pneumonia	Low
**Steroids (n=10)**									
Bronchiolitis									
Ahmad 2011	Pakistan	RCT	3-24 months	To determine the efficacy of steroids in bronchiolitis	90	Hospital Ward	Group 1 IV hydrocortisone 5mg/kg/dose q6h; Group 2 nebulized Beclomethasone 400-1000 ug q8h; Group 3 no steroids	No difference between groups in LOS (p=0.259), wheeze-free (p=0.40) or mean difference in RDAI (p=0.056)	Some
Mesquita 2009	Paraguay	RCT	2-24 months	To compare the efficacy of a single dose of oral dexamethasone in infants with moderate to severe bronchiolitis presenting to an ED	65	ED / A&E	Single dose oral dexamethasone 0.5mg/kg PO versus placebo in addition to standard of care (nebulized adrenaline)	No differences in RDAI (p=1.0), heart rate (p=0.4) and respiratory rate (p=0.3) and SpO2 (p=1.0) between groups after the 1st and 4th hours. No difference in hospitalization rate between groups (21 % vs. 25 %, p = 0.9, respectively)	Some
Qureshi 2015	Pakistan	RCT	2-24 months	To compare the mean duration of bronchiolitis in children receiving standard treatment with and without IV steroids	140	Hospital Ward	IV hydrocortisone 10mg/kg/day TID x 7 days	No difference between treatment and placebo groups for hospital LOS (5 vs 4.87 days, p=0.604), duration of wheezing (4.07 vs 4.26 days, p=0.535) or tachypnea (4.7 vs 4.5 days, p=0.471).	Low
Teeratakulpisarn 2007	Thailand	RCT	0.5-24 months	To examine the efficacy of a single intramuscular	174	ED / A&E	Dexamethasone (a single intramuscular	Decreased time to resolution of respiratory distress (HR 1.56; 95% CI, 1.14,2.13;	Low

				injection of dexamethasone in children hospitalized with acute bronchiolitis and followed up for 1 month after discharge			injection of 0.6 mg/kg) or placebo solution (an equivalent volume of saline) in a container of identical appearance	P<0.005), mean duration of symptoms of 11.8 hours (95% CI, 3.9,19.7; P<0.004), mean duration of oxygen therapy of 14.9 hours (95% CI, 5.3,24.4; P<0.003), and mean length of hospital stay of 13.4 hours (95%CI, 2.6,24.2; P<0.02) in dexamethasone group.	
Virk 2014	Pakistan	RCT	3-24 months	To compare the efficacy of prednisolone as an adjunct to conventional treatment or conventional treatment alone in patients of bronchiolitis	80	Hospital Ward	Oral prednisolone 2mg/kg/day in 3 divided doses	Decreased rhonchi on the third day of admission 67.5% vs 22.5%, p<0.0001), and shorter hospital LOS (4.72±1.17 days vs 5.72±1.5 days; p = 0.0014) in prednisolone group.	High
Zhang 2003	Brazil	RCT	<12 months	To assess short-term (and long-term) effect of prednisolone in hospitalized infants with bronchiolitis	52	Hospital Ward	1 mg/kg/day prednisolone x5 days	No difference in hospital LOS (6.0 vs 5.0 days, P = 0.7), duration of oxygen therapy (24.0 vs 24.0 h, P = 0.4) and time to clinical resolution (4.0 vs 4.0 days, P = 0.8)	Some
Croup									
Sumboonnanonda 1997	Thailand	RCT	2-37 months	To determine the beneficial effect of dexamethasone in children with moderate to severe infectious croup	32	Hospital Ward	0.5 mg/kg/d IM/IV dexamethasone x3 days	The dexamethasone group had significantly lower croup scores at 48 hour (p < 0.05), shorter hospital course (p < 0.005) and lower incidence of endotracheal intubation (p < 0.05) than the control group	Some
PJP Pneumonia									
Green 2008	South Africa	RCT	<18 months	To determine the efficacy and safety of adjunctive corticosteroid therapy in clinical Pneumocystis jiroveci pneumonia (PJP) in infants exposed to HIV infection	100	Hospital Ward	PO prednisone 2 mg/kg/day for 7 days.	43% better chance of survival in steroid vs. placebo group (hazard ratio (HR) 0.57, 95% confidence interval (CI) 0.30 - 1.07, p=0.08). No significant differences found with other parameters (oxygen requirement, time to recovery).	Low
Newberry 2017	Malawi	RCT	2-6 months	To determine whether early administration of adjuvant corticosteroids in addition to standard treatment reduces mortality in infants with vertically acquired HIV and clinically diagnosed PJP when co-infection with cytomegalovirus and other pathogens cannot be excluded	78	ED / A&E	Steroid regimen consisted of once daily oral prednisolone, 2 mg/kg for 7 days, then 1 mg/kg for 7 days, then 0.5 mg/kg for 7 days for a total of 21 days	Risk ratio of in-hospital mortality in the steroid group compared with the standard treatment plus placebo group was 0.53 [95% CI 0.29,0.97, p = 0.038].<br>The risk ratio of mortality at 6 months was 0.63 (95% CI 0.41, 0.95, p = 0.029).	Low
**Other medications (n=4)**									
Bronchiolitis									

Shang 2017	China	RCT	3-24 months	To evaluate the efficacy and safety of Laggera pterodonta in hospitalized children aged 3-24 months with acute bronchiolitis	133	Hospital Ward	Laggera pterodona mixture as tincture/syrup, q8h x 5 days	More children fulfilled the discharge criteria at 96 h and 120 h in the Laggera pterodontamixture group compared to the control group (97% vs 75.8%; P<0.001 and 98.5% vs 89.4%; P=0.03), although no difference at 72 h. Lower respiratory rate and oxygen saturation levels (p<0.001) in treatment group.	Some
Influenza									
Dawood 2016	El Salvador, Panama	RCT	0-9 years		688	Hospital Ward	Oseltamivir: 0-11mo, 3mg/kg/dose; >=12m: 30mg/dose if <=15kg, 45mg if 15-23kg; 60mg if 23-40kg, 75mg if>40kg. Every 12h for 10 doses.	No difference in median hospital LOS (3 days, IQR 2-4 vs. 5 days, IQR 3-7, p=0.22) or work of breathing (36 h, IQR 24-72 vs. 96 h, IQR 13-108, p=0.14) between oseltamivir versus placebo.	Some
Pneumonia									
Adinatha 2020	Indonesia	RCT	2-59 months	To evaluate the probiotic effectiveness as adjuvant therapy in childhood pneumonia compared to placebo	54	Hospital Ward	Probiotics (L. acidophilus, B. longum, Streptococcus thermophilus each 1x107 cfu/gr) granules 2 times a day for 5 days versus placebo	No difference on hospital LOS, duration of fever, retractions between two groups (p>0.05). However, decreased rales duration (p=0.037).	High
BecinaPaoloGene 2014	Philippines	RCT	2 months -4 years	To determine the efficacy of probiotics as adjunct therapy for pediatric community-acquired pneumonia	77	Hospital Ward	1 sachet of probiotics once a day for 7 days	No difference in heart rate, respiratory rate, temperature, presence of retractions or hospital LOS.	Some

Abbreviations: bCPAP: bubble continuous positive airway pressure; CI: confidence interval; IQR: interquartile range; IRR: incidence rate ratio; LOS: length-of-stay; RDAI: respiratory distress assessment instrument

Figure S1. Consensus judgements on risk of bias for each included study. Each risk of bias item is assigned a color-coded ranking; green color represents low risk of bias, yellow some concerns, and red high risk of bias.

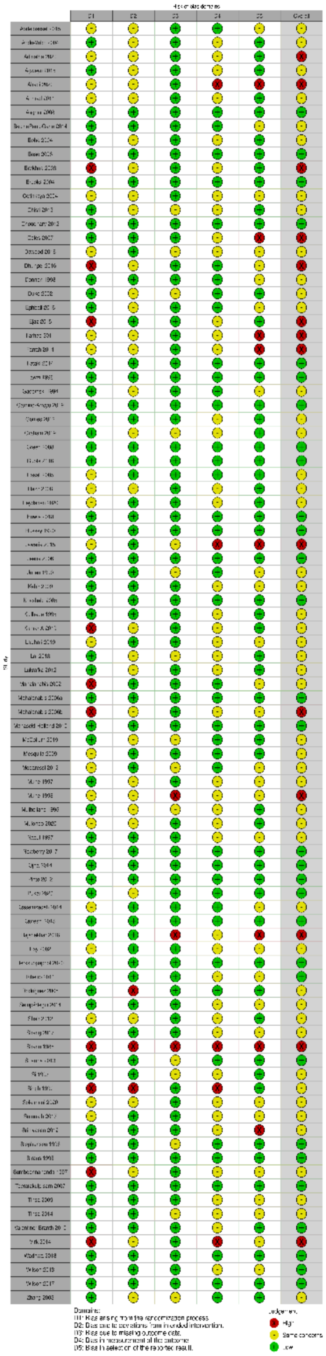

Figure S2. Summary of risk of bias assessments for included non-randomized studies according to the ROBINS-I tool.

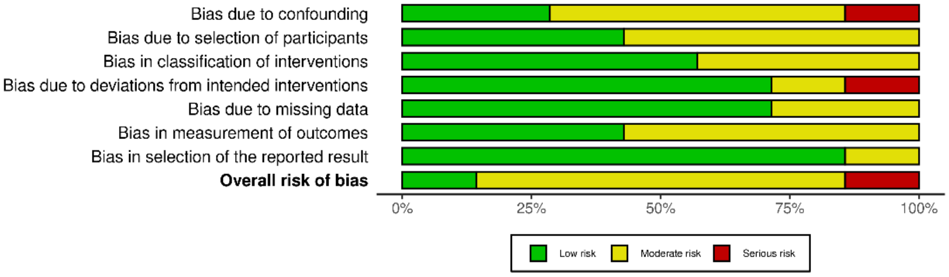

Figure S3. Consensus judgements using Cochrane ROBINS-I for each included nonrandomized study. Each risk of bias item is assigned a color-coded ranking; green color represents low risk of bias, yellow some concerns, and red high risk of bias.

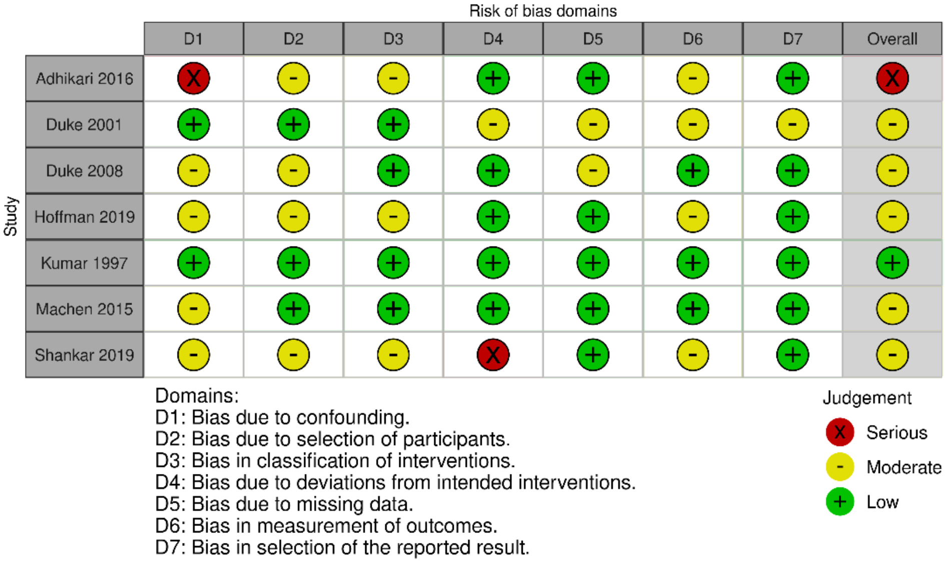

Supplement: Online Supplementary Document [file jogh-13-04065-s001.pdf]
